# Exploiting gene deletion fitness effects in yeast to understand the modular architecture of protein complexes under different growth conditions

**DOI:** 10.1186/1752-0509-3-74

**Published:** 2009-07-18

**Authors:** Roland A Pache, M Madan Babu, Patrick Aloy

**Affiliations:** 1 Structural and Computational Biology, Institute for Research in Biomedicine (IRB) Barcelona, c/Baldiri Reixac 10-12, 08028 Barcelona, Spain; 2 Life Sciences, Barcelona Supercomputing Center (BSC), c/Jordi Girona 29, 08034 Barcelona, Spain; 3 Systems Biology, MRC Laboratory of Molecular Biology, Hills Road, CB2 2QH Cambridge, UK; 4 Institució Catalana de Recerca i Estudis Avançats (ICREA), Passeig Lluís Companys 23, 08010 Barcelona, Spain

## Abstract

**Background:**

Understanding how individual genes contribute towards the fitness of an organism is a fundamental problem in biology. Although recent genome-wide screens have generated abundant data on quantitative fitness for single gene knockouts, very few studies have systematically integrated other types of biological information to understand how and why deletion of specific genes give rise to a particular fitness effect. In this study, we combine quantitative fitness data for single gene knock-outs in yeast with large-scale interaction discovery experiments to understand the effect of gene deletion on the modular architecture of protein complexes, under different growth conditions.

**Results:**

Our analysis reveals that genes in complexes show more severe fitness effects upon deletion than other genes but, in contrast to what has been observed in binary protein-protein interaction networks, we find that this is not related to the number of complexes in which they are present. We also find that, in general, the core and attachment components of protein complexes are equally important for the complex machinery to function. However, when quantifying the importance of core and attachments in single complex variations, or *isoforms*, we observe that this global trend originates from either the core or the attachment components being more important for strain fitness, both being equally important or both being dispensable. Finally, our study reveals that different isoforms of a complex can exhibit distinct fitness patterns across growth conditions.

**Conclusion:**

This study presents a powerful approach to unveil the molecular basis for various complex phenotypic profiles observed in gene deletion experiments. It also highlights some interesting cases of potential functional compensation between protein paralogues and suggests a new piece to fit into the histone-code puzzle.

## Background

Determining the fitness of an organism upon deletion of individual genes is a key strategy to decipher their function and relative contribution to survival. In the last years, several large-scale gene knock-out experiments in the budding yeast *Saccharomyces cerevisiae *identified genes which are essential for survival, and delivered quantitative fitness information for almost all inessential genes under a range of different growth conditions [1-6]. Although the effects of single gene knockouts have been analyzed in the context of binary protein-protein interaction networks [7,8], their interpretation with respect to protein complexes has not yet been systematically carried out. Such study is crucial to improve our understanding of living systems, simply because most major cellular processes, such as DNA transcription, translation, metabolism or replication, are not carried out by single proteins, but by dedicated molecular machines made of large protein assemblies.

Recently, two large-scale proteomics initiatives identified many novel macromolecular complexes in yeast consisting of up to several dozens of components [9,10]. With this new data it now becomes possible to interpret the results of gene deletion experiments in the light of a large set of protein complexes and their importance for cell survival.

We based our study on the set of 491 protein complexes, involving 1487 proteins, that Gavin *et al*. identified from over 2000 successful tandem affinity purifications [9]. In their study, the authors suggested a modular and hierarchical organization for the yeast cell machinery, where each complex is in reality a dynamic ensemble of complex variations, or *isoforms*. It is important to note that Gavin *et al*. [9] derived complex isoforms computationally and, although some have been proved to be biologically relevant, many could be ill-defined or mere artifacts from their genome-wide affinity purification screen. Complexes and isoforms are then composed of a mostly invariable set of proteins, which they defined as the complex core, and a number of peripheral proteins, the attachments, that complement and modulate the main complex function. This modular architecture of protein complexes in yeast has recently been supported by several types of proteomics data [11]. As the second prop of our study, we used fitness information of yeast single-gene deletion strains as determined by Steinmetz *et al*. for five major growth conditions of yeast, covering both fermentable (yeast extract peptone dextrose, YPD, and yeast extract peptone dextrose glycerol ethanol, YPDGE) as well as non-fermentable media (yeast extract peptone glycerol, YPG, yeast extract peptone ethanol, YPE, and yeast extract peptone lactate, YPL) [3].

In the last years, several studies used synthetic genetic interaction data, determined either through synthetic genetic arrays (SGAs) [12,13] or epistatic miniarray profiles (EMAPs) [14,15], to deduce functional relationships between gene pairs, identify sets of genes which function within the same complex or pathway and to predict the function of uncharacterized genes. Quantifying genetic interactions made it possible to identify sets of proteins acting together to perform a single function and provided insights into the functional organization of biological processes and their interdependencies [14]. One of the main areas of application for genetic interaction data is thus to discover sets of proteins which belong to the same pathway or complex. However, as sets of protein complexes in yeast have already been determined using tandem-affinity purification (TAP) data, and as Collins *et al*. [15] recently demonstrated that large-scale TAP data has a higher sensitivity at detecting proteins which belong to the same complex than genetic interaction data, we decided to integrate the TAP complexes data of Gavin *et al*. [9] with quantitative data of single gene deletions. More recently, Collins *et al*. [15] employed the EMAP approach to divide physical interactions into those in which the proteins function coherently and those where the proteins carry out distinct functions. They then used this separation of physical interactions to dissect protein complexes involved in yeast chromosome biology into functionally coherent modules [15]. As the complexes data of Gavin *et al*. already provides a separation into functionally coherent cores, modules and attachments [9], we did not have to use genetic interaction data to try to identify those modules. Instead, we focused on determining if and in which way the presence of genes in complexes and their modular components influences the fitness of yeast strains.

Several studies have provided the first hints that the presence of genes in protein complexes might affect strain fitness. For instance, Sarah Teichmann and colleagues [16,17] have demonstrated that proteins which are involved in important biological processes, such as transcription, translation and replication, are less dispensable than other genes, and that those proteins are often part of protein complexes (e.g. the RNA polymerase, the ribosome and the DNA polymerase). Recently, several studies have started to investigate the occurrence of essential genes in protein complexes [18-20]. In particular, Dezso *et al*. [18] studied the essentiality, functional role and subcellular localisation of proteins in the set of complexes defined by Gavin *et al*. from their first TAP experiment in 2002 [21]. As for those complexes, no modular architecture had been described, they defined highly coexpressed proteins of a complex as its core and showed that proteins in those cores often display the same deletion phenotype (i.e. essential or inessential). The authors then used this observation to classify complexes into essential and inessential ones [18]. Hart *et al*. [19] and Wang *et al*. [20], on the other hand, merged the raw TAP data of Gavin *et al*. [9] and Krogan *et al*. [10] to define their own sets of protein complexes using different clustering procedures. Based on their observation that essential genes tend to cluster in large complexes, they then suggested that essentiality is in many cases a product of complex function [19,20]. In contrast to those studies, we used the new complexes data of Gavin *et al*. [9] which, together with its description of the modular architecture of protein complexes based on the raw TAP data, allows us to investigate in detail the role of protein complex components, cores and attachments in establishing strain fitness. Importantly, we also use quantitative fitness data to demonstrate that many trends which we observe remain significant when looking only at the deletion effects of inessential genes.

According to the prevalent view, protein hubs (i.e. proteins with many interaction partners) tend to be more essential than non-hub proteins in interaction networks [22-25]. Although this so-called centrality-lethality rule had been questioned in the past [26,27], no clear conclusions could be extracted. Only very recently, Yu *et al*. [28] have presented clear evidence for a high-quality binary interaction network, constructed from Y2H data, that protein connectivity does not correlate with essentiality and argue that this discrepancy with earlier findings originates from biases towards essential and well-studied proteins in the original datasets used in those studies. Here we investigated this property specifically for proteins in large stable complexes. Pereira-Leal *et al*. [17] observed a trend that proteins belonging to multiple complexes seem to be more likely essential than proteins which are part of only one complex. However, they performed their analysis on the small set of complexes in the MIPS database [29], which show almost no overlap in their components (i.e. only 15 proteins are part of more than three MIPS complexes), as well as on the first generation of TAP data. The set of complexes defined by Gavin *et al*. [9] which we used to perform our analyses is much larger with many proteins belonging to multiple complexes and thus allows to test the statistical significance of observations.

Here, we systematically compare fitness information for genes which are part of complexes to those which are not, and investigate the distributions of essential and inessential genes within and across protein complexes in yeast. Moreover, we find convincing evidence that centrality in protein complexes does not correlate with essentiality and present the first attempt to quantify the importance of single complex isoforms, which we believe are the functional complex units, on strain fitness under different growth conditions.

## Results

### Genes in complexes show more severe fitness effects upon deletion

**Figure 1:**
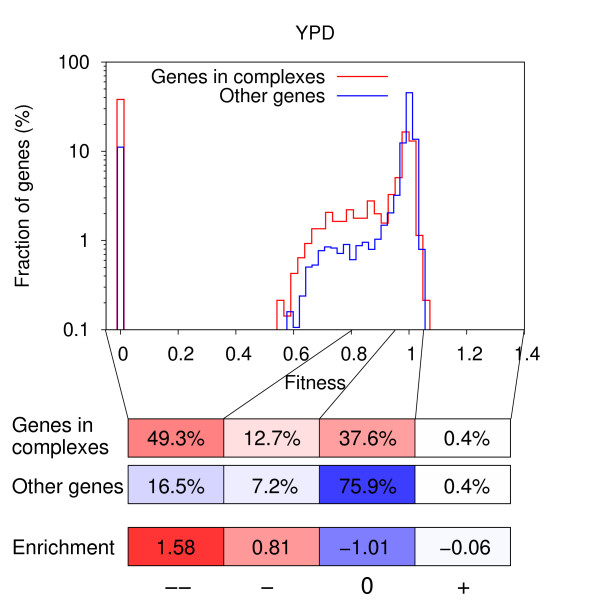
**Comparison of the fitness of yeast strains upon deletion of genes in complexes and other genes**. Distributions of strain fitness upon deletion of genes in complexes (red) and genes not part of complexes (blue) in YPD medium. Genes with a fitness of zero are essential. The fitness values of individual genes are partitioned into four categories: 'strong negative effect' (--), 'moderate negative effect' (-), 'weak or no effect' (0) and 'positive effect' (+). Different shades of red illustrate the percentage of genes in complexes (for which we have essentiality data) in the four fitness categories, with deep red corresponding to 100% (1404 genes). Different shades of blue illustrate the percentage of genes not in complexes (for which we have essentiality data) in the four fitness categories, with deep blue corresponding to 100% (3770 genes). Enrichments are given on a log_2_-scale.

As most processes in a cell are carried out not by single proteins, but by protein complexes, we first compared the fitness of yeast strains upon deletion of genes which are part of complexes to the fitness when deleting genes which are not. We partitioned the fitness values of individual genes into four categories: 'strong negative effect', 'moderate negative effect', 'weak or no effect' and 'positive effect', based on the distribution of all fitness values determined for a particular growth condition (see *Methods*). For YPD medium, the deletion of 49% of the genes in complexes leads to a strong negative fitness effect, whereas the same is true for only 17% of genes not present in complexes. Although the fraction of genes in complexes which lead to a moderate negative fitness effect upon deletion is considerably smaller with 13%, there is still a significant enrichment compared to the 7% of genes not in complexes (Fig. 1). Importantly, the enrichment of genes in complexes in the strong and moderate negative fitness effect categories is present in all growth conditions considered (see Additional file 1: Fig. S1) and is highly statistically significant (all p-values in the range [3.58 · 10^-120^, 7.37 · 10^-5^], one-sided Fisher's exact test).

To investigate whether the observed enrichment in the strong negative effect category originates only from the large fraction of essential genes (36% of genes in complexes are essential compared to only 10% of genes not part of complexes), we repeated the experiment excluding all essential genes from our calculations. As expected, the enrichment decreased in most media (see Additional file 1: Fig. S2), but it is still present and highly significant (all p-values in the range [3.95 · 10^-26^, 3.75 · 10^-9^], one-sided Fisher's exact test), meaning that the deletion of genes in complexes has overall a stronger negative effect on strain fitness than the deletion of genes not part of complexes.

In order to ensure that our findings are not restricted to the set of complexes defined by Gavin *et al*. [9], we applied the same analysis to the hand-curated set of 266 yeast complexes in the Munich Information Center for Protein Sequences (MIPS) database [29], as well as to the set of 547 complexes defined by Krogan *et al*. [10]. Our results hold with 52% and 17% of the genes in MIPS complexes leading to a strong and moderate negative effect in YPD medium, respectively (see Additional file 1: Fig. S3), compared to only 18% and 6% of genes not part of MIPS complexes (all p-values in the range [1.34 · 10^-117^, 6.88 · 10^-5^], one-sided Fisher's exact test). Considering only inessential genes (35% of the genes in MIPS complexes are essential compared to only 11% of the remaining genes), the enrichment for genes in MIPS complexes in the strong negative fitness effect category even increases (see Additional file 1: Fig. S4), while remaining significant (all p-values in the range [1.32 · 10^-47^, 1.83 · 10^-12^], one-sided Fisher's exact test). Also for the set of 547 Krogan complexes our results hold, as the enrichment of genes in Krogan complexes in the strong and moderate negative fitness effect categories, compared to genes not part of those complexes, although a bit lower are still present and significant, both when considering essential and inessential genes (see Additional file 1: Fig. S5; all p-values in the range [2.28 · 10^-46^, 7.26 · 10^-3^], one-sided Fisher's exact test), as well as when considering only inessential genes (see Additional file 1: Fig. S6; all p-values in the range [5.05 · 10^-11^, 5.45 · 10^-3^], one-sided Fisher's exact test).

Although both, the definition of protein complexes and the fitness data, come from high-throughput studies, we nevertheless considered the possibility that these datasets could be biased towards well-studied proteins. Such a bias could influence our analysis, as many well-studied proteins are part of important biological processes or pathways and might thus be enriched in essential genes. To assess this issue, we repeated the analysis, excluding the 153 complexes in the Gavin *et al*. set with a significant overlap to known complexes in the hand-curated MIPS database. We found that for the remaining 338 complexes, there is still a significant enrichment of genes in complexes in the strong negative effect category for all media (all p-values in the range [1.66 · 10^-73^, 3.56 · 10^-55^], one-sided Fisher's exact test). Excluding also all essential genes shows that the enrichment in the strong negative effect category remains significant (all p-values in the range [1.11 · 10^-20^, 1.88 · 10^-7^], one-sided Fisher's exact test) and thus does not originate only from the large fraction of essential genes (see Additional file 1: Table S1).

To make sure that the fitness data was also unbiased, we repeated the analysis described above, but excluding all known genes and keeping only those which are annotated as "Uncharacterized ORF". We found that our results hold with the enrichments of genes in complexes in the strong negative fitness effect category still being significant in all media (all p-values in the range [1.71 · 10^-5^, 2.26 · 10^-4^], one-sided Fisher's exact test). Excluding, in addition, also all essential genes, the observed enrichment of genes in complexes in the strong negative effect category remains significant in YPD and YPDGE medium (p-values = (1.60 · 10^-2^, 2.20 · 10^-2^), one-sided Fisher's exact test). The fact that the p-values are higher than in the original analysis and become insignificant for the non-fermentable media (p-values in the range [5.42 · 10^-2^, 1.32 · 10^-1^], one-sided Fisher's exact test), might well result from the fact that merely 1335 out of 5895 non-dubious genes in yeast are yet unknown (997 of them are inessential), and only 74 of them (62 inessential) are present in complexes, which greatly reduces the expressiveness of the statistical analysis (see Additional file 1: Table S2).

Although we have used a very stringent definition of "extensively studied protein" or complex (i.e. any protein that has ever been annotated or a complex that resembles any other complex in MIPS), our results still hold and we observe similar effects when considering only those complexes or proteins with little or no annotation, which demonstrates that they are not hampered because of any bias in the complexes data of Gavin *et al*. or in the fitness data determined by Steinmetz *et al*.

### Protein hubs do not show a higher degree of essentiality

**Figure 2:**
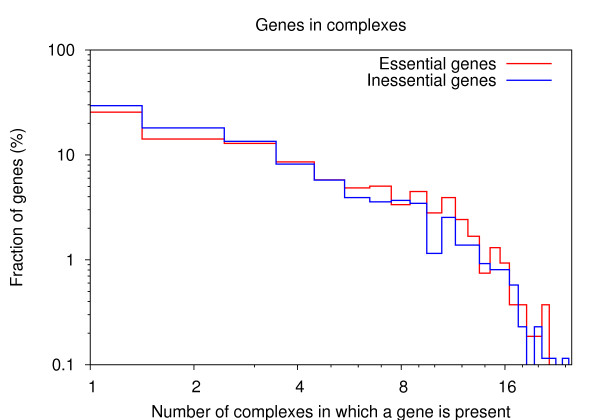
**Distributions of essential and inessential genes across complexes**. Distributions of the fraction of essential (red) and inessential genes (blue) which are present in a given number of complexes.

Proteins that belong to many complexes generally have significantly more interaction partners than those present in only one or a few complexes (see Additional file 1: Fig. S7), and thus correspond to connection hubs in many protein-protein interaction network representations. Based on the current view on binary interaction networks that hubs tend to be more essential than non-hub proteins [22-25], one would expect the fraction of essential genes which are present in many complexes to be significantly higher than the fraction of inessential genes. However, we found that the distribution of essential genes across complexes is not significantly different from that of inessential genes (p-value = 0.34, one-sided Fisher's exact test), with both distributions showing an exponential decay in the fraction of genes with increasing number of complexes in which they are present (Fig. 2). This comparison of the distributions shows that genes which are part of many complexes (i.e. the hubs) are not more likely to be essential than genes which are present in only one or a few complexes (i.e. non-hub proteins), independent of any cutoff chosen for distinguishing hubs in protein complexes from non-hub proteins. Moreover, the fraction of essential genes which are part of more than 12 complexes (top fifth percentile), and which could thus be defined as hubs in protein complexes, is with 6% only slightly higher than the fraction of inessential genes, 5%, and, compared to the fractions of essential and inessential genes part of only one complex (bottom fifth percentile), this difference is not significant (p-value = 0.25, one-sided Fisher's exact test). We also binned the genes into two sets, those which are part of more than 12 complexes (top fifth percentile) vs. all other genes, again finding no significant difference (p-value = 0.29, one-sided Fisher's exact test).

Thus, in our analyses, shared components between many complexes (i.e. the hubs) are not more likely to be essential than non-hub proteins.

**Figure 3:**
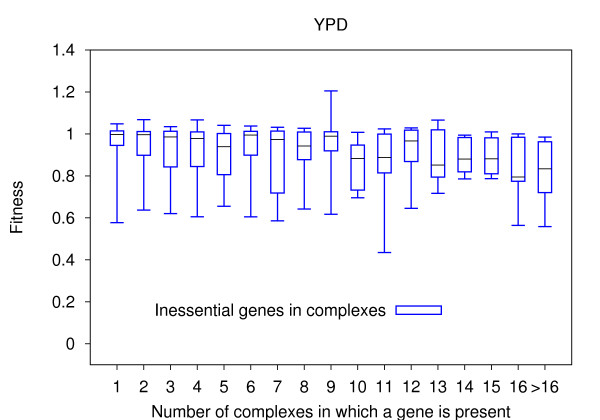
**Fitness of yeast strains upon deletion of inessential genes present in multiple complexes**. Box-and-whisker plots of strain fitness upon deletion of inessential genes which are part of multiple complexes, measured in YPD medium. Start and end of the boxes indicate the first and third quartile of the fitness distribution of inessential genes present in a given number of complexes, and whiskers denote the respective minimum and maximum fitness values. The medians of the respective distributions are shown as black bars. As only 21 inessential genes are present in more than 16 complexes, we grouped them together.

When looking at the quantitative fitness data for inessential genes, we further observed that strain fitness upon deletion of an inessential gene does not depend on the number of complexes in which the gene is present either (Fig. 3). The results are consistent in all five media considered (see Additional file 1: Fig. S8), suggesting that the deletion of protein hubs in complex networks does not have a more severe effect than deletion of non-hub proteins.

Protein abundance might have an effect on interaction properties and thus influence our analyses. To control for this possibility, we checked whether the abundance of yeast proteins in general, or of only those proteins which are part of complexes, is correlated with fitness data. As reported by Gavin *et al*. for the protein complexes data used in this study, their tandem affinity purification procedure favoured more abundant proteins, but, nevertheless, they still detected some proteins with low abundance [9]. Analysis of the yeast protein abundance data originating from Ghaemmaghami *et al*. [30] revealed that, although significant (all p-values < 10^-4^, Monte Carlo permutation test), the correlation between strain fitness upon deletion of protein-coding genes and protein abundance is very small, both when considering all yeast proteins (*γ *≈ -0.12, see *Methods*), and when taking only those proteins into account that are part of complexes (*γ *≈ -0.08; see Additional file 1: Fig. S9). When comparing the abundance distributions of essential and inessential genes in complexes, we found that the average abundance of essential genes (21,182 +/- 84,818) is only slightly higher than the one of inessential genes (19,616 +/- 52,782) with very large standard deviations (p-value = 0.04, two-sided Mann-Whitney U test). Thus, even if protein abundance correlates with interaction properties, as the correlation between abundance and essentiality is very small, and as we do not observe a significant correlation between centrality and essentiality in protein complexes, we can conclude that protein abundance does not significantly influence our analyses.

We could not repeat the analyses for the curated set of yeast complexes in the MIPS database [29] since, by definition, those complexes show almost no overlap in their components (only 15 proteins are present in more than three MIPS complexes). The same is true for the Krogan complexes, as the clustering procedure used by Krogan *et al*. to define the complexes from the raw TAP data does not allow proteins to belong to several complexes [10].

### Genes within the modular components of complexes show similar fitness effects

**Figure 4:**
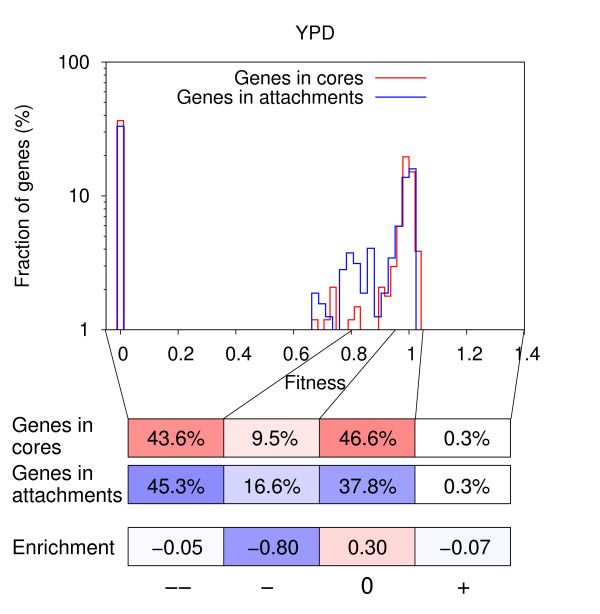
**Comparison of the fitness of yeast strains upon deletion of genes unique to complex cores and genes unique to attachments**. Distributions of strain fitness upon deletion of genes only present in cores (red) and genes only present in attachments (blue) in YPD medium. Genes with a fitness of zero are essential. The fitness values of individual genes are partitioned into four categories: 'strong negative effect' (--), 'moderate negative effect' (-), 'weak or no effect' (0) and 'positive effect' (+). Different shades of red illustrate the percentage of genes in cores (for which we have essentiality data) in the four fitness categories, with deep red corresponding to 100% (337 genes). Different shades of blue illustrate the percentage of genes in attachments (for which we have essentiality data) in the four fitness categories, with deep blue corresponding to 100% (320 genes). Enrichments are given on a log_2_-scale.

In their genome-wide study of protein complexes in yeast, Gavin *et al*. defined 5979 complex variations, which they termed complex isoforms, and suggested a modular architecture for protein complexes: a complex consists of a core of proteins which determine the basic machinery, invariable in most isoforms, and certain attachment proteins, depending on the cellular conditions, that complement and modulate the main function [9]. Both core and attachment proteins are equally necessary for complexes to function. However, each complex exists in several different variations (i.e. isoforms) in a cell, with only the core proteins being common to most of them. Thus, if a gene encoding a core protein is deleted, it affects many more complex isoforms than if a gene coding for an attachment protein is deleted (which is part of only one or a few isoforms). One could consequently hypothesize that genes within cores might show a stronger negative fitness effect upon deletion than genes in attachments. When comparing the sets of core and attachment proteins, we found a substantial overlap of 791 genes between the 1148 non-redundant core genes and the 1130 non-redundant attachment genes (i.e. core components in one complex might well be attachments in another). We thus excluded those overlapping genes, when testing for an enrichment of genes with a negative fitness effect upon deletion in cores. For YPD medium, 44% of genes unique to cores are in the strong negative effect category, while the same is true for about an equal fraction of genes, 45%, that are unique to attachments (Fig. 4). The same holds for all five media considered (see Additional file 1: Fig. S10), with no significant enrichments (all p-values in the range [0.69,1.0], two-sided Fisher's exact test). The slight enrichment for genes in attachments in the moderate negative effect category that we found here turned out to be statistically significant only in YPD, YPDGE and YPG medium (p-values in the range [7.48 · 10^-3^, 1.37 · 10^-2^], two-sided Fisher's exact test), but not in YPE and YPL medium (p-values = (0.53,0.81), two-sided Fisher's exact test). As many genes are present both in cores and attachments, and because we observe no significant difference between the fraction of genes unique to cores and the fraction of genes unique to attachments that lead to a strong negative fitness effect upon deletion, we conclude that, in general, core and attachment components are equally important for the complex cellular machinery.

### Single isoforms feature distinct fitness patterns across media

As described before, complex isoforms consist of core components and certain attachment proteins, depending on the given cellular conditions, and are thus the functional biological entities [9]. To be able to quantify the importance of single isoforms and their cores and attachments under different growth conditions, we explored the possibility of assigning a single fitness value to each isoform and to its core and attachment proteins, which would represent the fitness of the respective modular components. This is, to test the coherence of fitness values within each set of genes across the five media studied and check whether it is possible to convert experimental data obtained for individual genes into a global figure representative of a particular complex core, set of attachments or isoform. As the coherence highly depends on the size of the given gene set, we first employed a size-correction procedure (see *Methods*) which ensures that the different fractions of coherent isoforms, cores, attachments and MIPS complexes we determined are comparable. Then we computed the coherence based on raw fitness values, as well as based on fitness categories (i.e. strong negative, moderate negative, weak or no effect and positive), respectively. We found only 26–36% of the isoforms, 38–50% of isoform cores and 25–33% of isoform attachments to be coherent, as well as 41–49% of MIPS complexes and 33–41% of Krogan complexes. Thus, we concluded that for the majority of them, one cannot simply assign the most prevalent fitness category of the given gene set, as the fitness values of the individual genes can differ too much. So, we used the average fitness value of the given set of genes, as it represents the expected fitness of a yeast strain when deleting a random gene of the respective isoform, core or attachments. This measure encompasses all individual fitness values of a given set and thus provides a more justifiable measure for the fitness of whole groups of genes.

**Figure 5:**
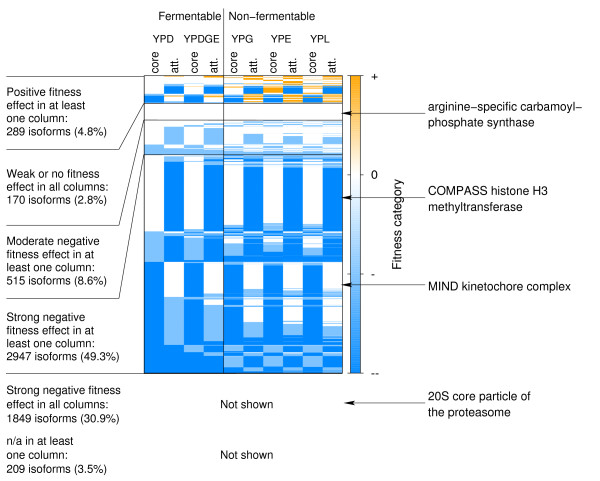
**Fitness of the complex core and attachments of single isoforms across different growth conditions**. Expected fitness effects upon deletion of a random component of the given core or set of attachment proteins for all 5979 isoforms across the two fermentable and the three non-fermentable media considered. The fitness values are partitioned into four categories: 'strong negative effect' (--/blue), 'moderate negative effect' (-/light-blue), 'weak or no effect' (0/white) and 'positive effect' (+/orange). Each line represents the fitness profile of a given isoform, treating the core and the attachments (att.) separately. 'n/a': the expected fitness effect is unknown due to a lack of quantitative fitness information for the genes in the respective core or attachments. When grouping the fitness profiles, we gave priority to n/a, positive, strong negative and moderate negative fitness effect in that order. Arrows indicate isoform fitness profiles of complexes given as examples in the main text.

The analysis of individual isoforms then revealed that the general trend of genes in cores and attachments displaying similar fitness effects upon deletion (see above and Fig. 4) is in reality the net result of either the core components or the attachments of a given isoform being more important for strain fitness, both being equally important or both being dispensable in all five media considered (Fig. 5; see Additional file 2: Fitness of cores, attachments). The contrary effects observed for some isoforms, that either the deletion of core or of attachment components leads to worse strain fitness, cancel each other out in the more general analysis depicted in Fig. 4. When looking at single isoforms, however, it becomes apparent that sometimes the core components are more important for strain fitness and sometimes the attachments, while for other isoforms both types of components are equally important or even dispensable. For example, the latter include isoforms of the arginine-specific carbamoyl-phosphate synthase complex, which takes part in arginine biosynthesis (not required in amino-acid rich media), while for all isoforms of the 20S core particle of the proteasome, which represents the main character in the protein degradation machinery, both core (i.e. different alpha- and beta-type subunits) and attachment components (e.g. regulatory subunits of the 26S proteasome) are equally important for cell survival. On the other hand, for isoforms of the MIND kinetochore complex, necessary for sister chromatid segregation during mitosis and meiosis, the core consists of essential components which join kinetochore subunits contacting DNA to those contacting microtubules and is thus more important for strain fitness than the attachments which merely contain non-essential kinetochore proteins. By contrast, for isoforms of the complex of proteins associated with Set1 (COMPASS) histone H3 methyltransferase, involved in transcriptional regulation, the attachments are more important, as they are, for example, also part of the cleavage and polyadenylation factor, a complex involved in RNA polymerase II transcription termination, or contain the ubiquitin hydrolase Doa4/Ubp4 which is required for ubiquitin recycling from ubiquitinated proteins bound to the proteasome.

**Figure 6:**
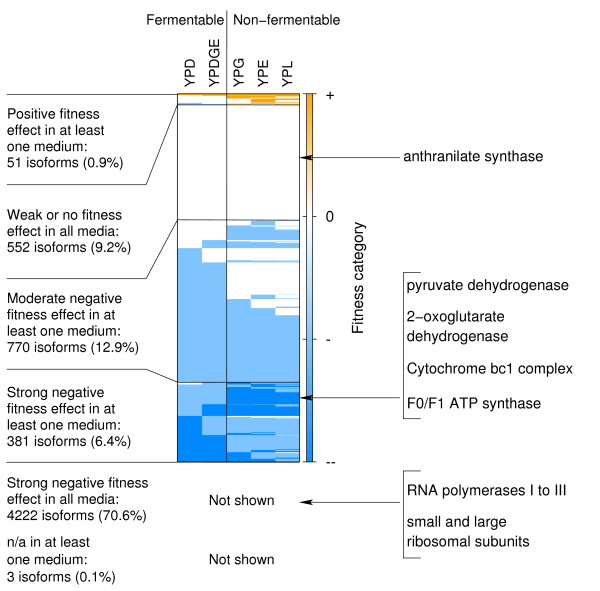
**Fitness of complex isoforms across different growth conditions**. Expected fitness effects upon deletion of a random gene of whole isoforms across the two fermentable and the three non-fermentable media considered. The fitness values are partitioned into four categories: 'strong negative effect' (--/blue), 'moderate negative effect' (-/light-blue), 'weak or no effect' (0/white) and 'positive effect' (+/orange). Each line represents the fitness profile of a given isoform. 'n/a': the expected fitness effect is unknown due to a lack of quantitative fitness information for the genes in the respective isoform. When grouping the fitness profiles, we gave priority to n/a, positive, strong negative and moderate negative fitness effect in that order. Arrows indicate isoform fitness profiles of complexes given as examples in the main text.

When investigating the expected fitness effect upon deletion of a random gene of whole isoforms, we found distinct patterns of expected strain fitness across the five growth conditions considered (Fig. 6; see Additional file 2: Fitness of whole isoforms). The majority of isoforms (71%) show a strong negative expected fitness effect upon deletion of a random component in all five media. Those include isoforms of the RNA polymerases I to III, which are necessary for all transcription processes in a yeast cell, and isoforms of both the small and the large ribosomal subunit, required for translation of messenger RNAs to proteins. Another 5% of the isoforms display a moderate negative effect upon deletion of a random component, and 10% of the isoforms seem to be dispensable in all five growth conditions. The latter include, for instance, isoforms of the anthranilate synthase complex which catalyzes the initial step of tryptophan biosynthesis (unnecessary in amino acid-rich media). Deletion of a random component of the remaining 14% of the isoforms leads to different fitness effects, depending on the respective growth condition. For instance, when comparing fermentable and non-fermentable media, we found several complex isoforms that are more important for cell survival in the non-fermentable media (i.e. YPG, YPE and YPL), in which yeast has to rely on aerobic respiration because of a lack of glucose. Those isoforms cover key elements of the respiratory pathway: the Pyruvate dehydrogenase complex, which transforms pyruvate into Acetyl CoA, the 2-oxoglutarate dehydrogenase complex, an enzyme of the tricarboxylic acid cycle, the Cytochrome bc1 complex, which is part of the electron transport chain and participates in establishing a proton gradient across the mitochondrial inner membrane, and the F0/F1 ATP synthase, which finally uses that gradient for the generation of ATP.

**Figure 7:**
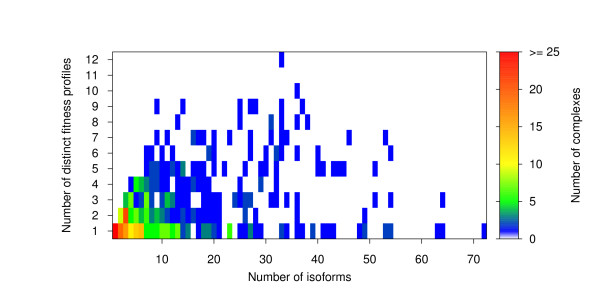
**Comparison of the number of isoforms and the number of distinct isoform fitness profiles for each complex**. Comparison of the number of isoforms and the number of distinct isoform fitness profiles for all 491 complexes. The number of complexes is indicated by a color scheme.

In order to find out whether the isoforms of individual complexes all have the same or different fitness profiles, we compared for each complex the number of isoforms and the number of distinct isoform fitness profiles (Fig. 7). This comparison revealed that, for 253 (52%) of the complexes, all isoforms have the same fitness profile and that only 9% of the complexes feature more than five distinct isoform fitness profiles. For 91 of the 253 complexes (36%), the fact that they feature only one fitness profile can easily be explained, as they have only one isoform. Notably, there exists only a moderate positive correlation between the number of isoforms and the number of distinct profiles (*γ *≈ 0.48 (see *Methods*), p-value < 10^-4^, Monte Carlo permutation test), because even when a complex has many isoforms, they can still exhibit the same fitness profile. For instance, the RNA polymerase II and the Translation initiation factor eIF3 complex have 64 and 63 isoforms, respectively, but all isoforms share the same fitness profile (strong negative effect in all media).

### Significantly more isoforms than expected contain no essential genes

Considering the distribution of essential genes across the 5979 isoforms, we found out that 74% of the isoforms contain at least one essential gene. As essential genes are enriched in complexes, it is indeed striking that 1539 isoforms (26%) include no essential gene at all (*p - value *< 10^-4^, Monte Carlo permutation test). We considered the possibility that this observation could, at least partially, be explained by direct backups in the form of duplicate (i.e. paralogous) genes, and searched for duplicates in the yeast genome (see *Methods*). We then checked the number of isoforms without essential genes for which a duplicate exists for every component. We found duplicates for 1562 genes in the yeast genome (27%) and discovered that 211 of the 1539 complex isoforms which contain no essential gene (14%) have duplicates for every component, which is statistically significant (p-value = 5.04 · 10^-61^, one-sided Fisher's exact test). Thus, for a significant number of isoforms without essential genes there exist duplicates in the yeast genome for every component, which might explain the lack of essential genes in those isoforms.

## Discussion

Based on the large set of protein complexes in yeast that Gavin *et al*. recently identified [9], we compared the fitness of yeast strains upon deletion of genes which are part of complexes to those which are not, and found out that there exists a significant enrichment of genes with a strong negative or moderate negative effect on strain fitness upon deletion in complexes. This enrichment, which is independent of the respective growth condition, could be explained by the fact that most processes in a cell are carried out not by single proteins, but by protein complexes and thus, knocking-out only one protein can damage the whole molecular machine. The work of Sarah Teichmann and colleagues [16,17] supports this explanation by demonstrating that proteins which are involved in important biological processes, such as transcription, translation and replication, are less dispensable than other genes, as well as more conserved in evolution and often part of protein complexes (e.g. the RNA polymerase, the ribosome and the DNA polymerase). Since most proteins in a cell spend at least part of their time in a complex with other proteins, it is worth clarifying that the study of Gavin *et al*. mainly captured stable multi-protein complexes, which they termed molecular machines. The components of these molecular machines usually spend most of their life forming part of the complex and have no function in isolation. It has been estimated that there are some 800 of these stable complexes in yeast [9], containing about 2,400 proteins, which would leave some 3,000 proteins free, even if they transiently associate with other proteins or complexes.

Although it has been already suggested that essential genes are enriched in complexes [18,19,21], our findings show that the enrichment of genes with a strong negative fitness effect upon deletion in complexes does not solely originate from those essential genes, but remains significant when considering only inessential genes. Application of the same analyses to the smaller, but hand-curated, set of yeast complexes in the MIPS database [29] and the large set of protein complexes defined by Krogan *et al*. [10] revealed that our results are not restricted to the set of complexes defined by Gavin *et al*., but actually represent a more general finding.

Concerning the on-going debate whether there exists a correlation between protein centrality and essentiality [17,22-27], only very recently, Yu *et al*. [28] have presented clear evidence for a high-quality binary interaction network, that protein centrality (or hubness) does not correlate with essentiality and argue that this discrepancy with earlier findings originates from biases towards essential and well-studied proteins in the original datasets used in those studies. Here we investigated this property specifically for proteins in large stable complexes. We discovered that the distribution of essential genes across complexes is not significantly different from the distribution of inessential genes. In fact, the fraction of essential genes which are part of many complexes is not significantly higher than the fraction of inessential genes. Importantly, when considering quantitative fitness data instead, we found that strain fitness upon deletion of an inessential gene is also independent of the number of complexes in which the gene is present, supporting our observation. Thus, complementing the recent findings of Yu *et al*. [28], our study provides clear evidence that hubs in protein complexes are neither more likely to be essential, nor do they tend to show more severe fitness effects upon deletion than non-hub proteins.

Gavin *et al*. suggested that protein complexes in yeast have a modular architecture with each complex consisting of a core of proteins found in most complex variations (i.e. complex isoforms) and certain attachments depending on the particular cellular conditions [9]. They also suggested that complex isoforms are most likely the functional forms of complexes, representing slight variations on the same molecular machine, and that core and attachment proteins are equally important to fulfill the biological functions.

Here, we tested whether gene deletion experiments would support this view or rather highlight a hierarchy, in terms of functional essentiality, among complex components. Our results indicate that the fraction of genes in the different fitness categories is virtually identical for genes in cores and genes in attachments across all growth conditions considered, placing them on the same level of importance within the complex hierarchy. When excluding all genes from the analysis which can be present both in cores and attachments, there is still little to no enrichment in the strong negative fitness effect category, and the slight enrichment for genes in attachments in the moderate negative effect category that we observed is not statistically significant in all media. We thus conclude that, in general, attachment proteins are equally important as core components for the complex machinery to function.

As complex isoforms represent the biological entities which act as molecular machines to fulfill particular tasks in a cell, we investigated the importance of single isoforms and their core and attachment components under different growth conditions. In contrast to previous studies [18,19], which did not consider quantitative fitness data, we found out that the majority of those gene sets are not coherent in terms of the fitness values of their components. This discrepancy can be explained by the different types of data used in those studies. Specifically, Dezso *et al*. based their analysis on the set of complexes originating from the first TAP study of Gavin *et al*. [21], performed in 2002, and defined their own set of core proteins for each complex based on coexpression data [18]. Hart *et al*., on the other hand, merged the raw TAP data of Gavin *et al*. [9] and Krogan *et al*. [10] to define their own set of protein complexes [19]. More importantly, both Dezso *et al*. and Hart *et al*. only compare the fractions of essential and inessential genes in each complex or core, but they do not consider quantitative fitness data and treat all inessential genes equally, independent of whether deleting them leads to a negative, no or positive fitness effect. The fact that complex isoforms, cores and attachments are not coherent when considering quantitative fitness data shows, that, although interacting proteins have more similar fitness effects upon deletion than random pairs of proteins [31], the fitness values of the individual components of isoforms, cores and attachments can still differ significantly. Indeed, we discovered that the global trend of genes in cores and attachments displaying similar fitness effects upon deletion is actually the net result of either the core components or the attachments of a given isoform being more important for strain fitness, both being equally important or both being dispensable. Thus, the general effect that we observed when comparing the importance of genes in cores to genes in attachments originates from a combination of several effects that only become apparent when looking at single complex isoforms.

**Figure 8:**
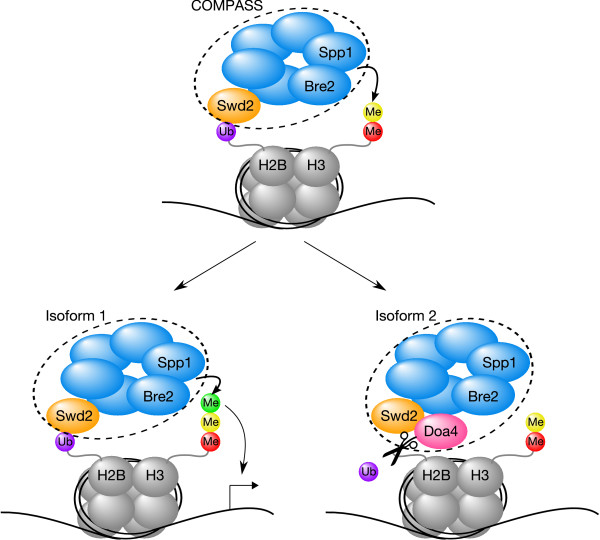
**Proposed model for the regulation of histone trimethylation by COMPASS through the recruitment of the attachment protein Doa4**. The COMPASS methyltransferase protein complex methylates lysine 4 at histone H3 (H3-K4), regulated by ubiquitination of lysine 123 at histone H2B (H2B-K123) [32-35]. H3-K4 can be mono-, di- and tri-methylated and COMPASS is required for all three levels of methylation, while only tri-methylated H3-K4 leads to the activation of gene transcription [39,40]. The two COMPASS components Spp1 and Bre2 are required for tri-methylation activity of the complex [40], but both belong to the core of COMPASS (shown in blue) and are present in all isoforms of the complex. The attachment protein Swd2 (orange) mediates the cross-talk between H2B-K123 monoubiquitination and H3-K4 di- and trimethylation [41]. Some isoforms of COMPASS contain the ubiquitin hydrolase Doa4 (magenta) as an attachment, and we thus propose that the addition of the third methyl group (green) to di-methylated H3-K4 by COMPASS is regulated by the recruitment of Doa4 to the complex. Removal of ubiquitin (purple) from H2B-K123 by Doa4 (indicated with scissors) would disrupt the association of Swd2 (orange) with chromatin (histones shown in grey with a black DNA string wrapped around), inhibiting H3-K4 trimethylation.

A particularly intriguing example (Fig. 8), where the attachments are more important for strain fitness (i.e. deletion of attachments leads to a negative fitness effect, while deletion of core components does not seem to affect strain fitness), is the COMPASS methyltransferase protein complex which methylates lysine 4 at histone H3 (H3-K4), regulated by ubiquitination of lysine 123 at histone H2B (H2B-K123) [32-35]. The last years have provided many new insights into the complex process of epigenetic regulation of transcription by covalent histone modifications [36-38], and the COMPASS complex represents a key player in the establishment of this so-called 'histone-code'. It has been found that H3-K4 can be mono-, di- and tri-methylated and that the COMPASS complex is required for all three levels of methylation [39,40]. However, Santos-Rosa *et al*. discovered that tri-methylated H3-K4 is present only at active genes and thus suggested that there exists a mechanism which regulates the addition of a third methyl group by the COMPASS complex [39]. Schneider *et al*. hypothesized that this regulation could be mediated by the presence or absence of the two COMPASS components Spp1/Cps40 and Bre2/Cps60, as they observed that these two subunits are required for tri-methylation activity of the complex [40], but we found both components to belong to the core of COMPASS and to be actually present in all isoforms of the complex. Recently, Lee *et al*. [41] demonstrated that Swd2/Cps35, an attachment of the COMPASS complex, mediates the cross-talk between H2B-K123 monoubiquitination and H3-K4 di- and trimethylation. They found out that the association of Swd2/Cps35 with chromatin, dependent on H2B-K123 ubiquitination, allows COMPASS to di- and trimethylate H3-K4, leading to gene activation [41]. On the other hand, we discovered that some isoforms of the COMPASS complex contain the ubiquitin hydrolase Doa4/Ubp4 as an attachment which, although it is not an essential gene like Swd2/Cps35, also leads to a negative fitness effect upon deletion. We thus propose that the addition of the third methyl group to di-methylated H3-K4 by COMPASS is regulated by the recruitment of Doa4/Ubp4 to the complex, representing a potential new piece to complete the histone-code puzzle. Removal of ubiquitin from H2B-K123 by Doa4/Ubp4 would disrupt the association of Swd2/Cps35 with chromatin, inhibiting H3-K4 trimethylation (Fig. 8). Although this is only a hypothesis which requires experimental validation, it is supported by the fact that Ubp8, a remote paralog of Doa4/Ubp4, has already been shown to be responsible for Spt-Gcn5-acetyltransferase (SAGA) complex mediated deubiquitination of H2B-K123 [41-45]. If our hypothesis is correct, Doa4/Ubp4 would as an attachment of COMPASS, in the same way as the association of Ubp8 to SAGA, result in one complex performing two posttranslational modification functions (i.e. methylation and deubiquitination).

When determining the expected fitness effect upon deletion of a random gene of whole isoforms, we found distinct patterns of expected strain fitness across the five growth conditions considered. More than two thirds of the isoforms show a strong negative expected fitness effect upon deletion of a random component in all five media. This could signify that most complex variations mediate cellular functions that are important for strain fitness independent of the growth conditions. Our results indicate that those isoforms which we observed to be dispensable in all five media are most probably required for cell survival in other growth conditions not considered in our analysis [6,46]. Furthermore, we found multiple isoforms which cover different key elements of the respiratory pathway to be more important in the non-fermentable media, where yeast has to use aerobic respiration for metabolism. This demonstrates that our approach for quantifying the importance of protein complex variations is able to detect those media-specific effects.

Comparing the number of isoforms and the number of distinct isoform fitness profiles for each complex, we observed that, for more than half of the complexes, all isoforms of the respective complex show the same fitness profile across the five media. Additionally, as there exists only a moderate positive correlation between the number of isoforms and the number of distinct fitness profiles, we suggest that many cellular conditions, for which the different isoforms of a complex get assembled, are internal states of the cell which are independent of the given carbon source.

Finally, by investigating the distribution of essential genes across isoforms, we found out that 74% of the isoforms have at least one essential gene. The lethal effect on the yeast strain when deleting one of those genes thus might originate from the given isoform not being able to fulfill its particular task in the cell. On the other hand, it has recently been observed for a set of 390 protein complexes in yeast, that essential genes are absent in significantly more complexes than expected [19]. Indeed, as essential genes are enriched in complexes, it is striking that 27% of the isoforms have no essential gene. A possible explanation for this could be the occurrence of compensatory effects with either direct backups for all genes in the respective isoform via duplicates, which we actually found to be the case for a significant fraction of those isoforms (14%), or alternative protein assemblies that are similar enough to provide the same functionality in the cell. Those compensatory effects would then leave more room for evolution-driven mutations to adapt the complex machinery of the cell to different environmental conditions.

## Conclusion

We have shown in this study how the integrated analysis of gene deletion fitness data and complex modular architecture can be a powerful approach to unveil the molecular bases responsible for some unexpected phenotypic profiles for a given knock-out. The challenge is now to extend these analyses to higher eukaryotes, and to develop computational models able to predict the functional behaviour, under different nutritional conditions, upon single or double-gene deletions in those organisms for which data is scarce or unavailable.

## Methods

### Databases of protein complexes in yeast

The current study is based on the large set of 491 protein complexes and 5979 isoforms (i.e. complex variations) that Gavin *et al*. recently identified in yeast from over 2000 successful tandem-affinity purifications [9] (see Additional file 3: Gavin complexes and Gavin complex isoforms). To ensure that our findings for protein complexes are not restricted to the set of complexes defined by Gavin *et al*., we applied the same analyses to the hand-curated set of 266 yeast complexes in the MIPS database [29] (see Additional file 3: MIPS complexes), which is often used as a 'gold standard'. Moreover, we also repeated the analyses for the large set of 547 yeast protein complexes that Krogan *et al*. [10] defined from tandem-affinity purification data (see Additional file 3: Krogan complexes). As the modular architecture of protein complexes described by Gavin *et al*. is neither available for the MIPS nor for the Krogan complexes, we could not repeat those analyses which depend on the description of such an architecture. However, we performed the controls using the MIPS and Krogan complexes, whenever possible.

### Quantitative fitness data for inessential genes

We used quantitative fitness data from the yeast gene deletion study conducted by Steinmetz *et al*., which provides fitness information for 4218 non-dubious inessential genes across nine different media [3] (see Additional file 3: Quantitative fitness data). The high-throughput nature of the screen ensures that the data is not biased towards extensively-studied genes.

In this study, we considered only the five major media: YPD (2% yeast extract, 1% Bacto-peptone and 2% glucose), YPDGE (2% yeast extract, 1% Bacto-peptone, 0.1% glucose, 3% glycerol and 2% ethanol), YPG (2% yeast extract, 1% Bacto-peptone and 3% glycerol), YPE (2% yeast extract, 1% Bacto-peptone and 2% ethanol) and YPL (2% yeast extract, 1% Bacto-peptone and 2% lactate). This is because in these media the organism is able to grow exponentially, matching thus the conditions used in Gavin *et al*. [9] to define the complexes. Although other exciting work on gene deletion fitness has been recently reported [4-6], we decided not to include these data in our study because these studies mostly considered severe stress conditions, which makes unclear whether the composition of complexes in those media might differ from their composition under exponentially growing conditions used in Gavin *et al*. [9]. Another advantage of using the five major media reported in Steinmetz *et al*., [3] is that, for these conditions, the yeast gene deletion screen experiments were performed twice, which allowed the authors to check for reproducibility of the results. After confirming that both time-course experiments indeed report similar fitness values for the different deletion strains (see Additional file 1: Fig. S11), we took the average fitness value from time-course one and two as the quantitative fitness of the respective deletion strain. In case the strain fitness could only be measured in one time-course, we used that measurement directly.

The negative growth rate for the YOL139C deletion strain, measured in time-course two for the medium YPL, is only an artifact from the fitting of regression lines to the logarithm of the hybridization intensities, which Steinmetz *et al*. employed to calculate fitness values for each deletion strain, and as such, we treated this negative growth rate as zero.

### Determination of essential genes

Genes of homozygous diploid yeast strains with zero measurements on the hybridization array, used in the molecular barcode technique employed by Steinmetz *et al*., are potentially essential for growth in YPD medium [3]. As they could also be measurement errors, we followed the approach of Gu *et al*. [47] by taking the intersection of three lists of genes, namely the two lists of genes with zero measurements of time-course one and two from Steinmetz *et al*. [3] and the list of essential genes reported by Giaever *et al*. [2], as a confident list of essential genes for this work (956 non-dubious genes; see Additional file 3: Essential genes). As all yeast strains were grown on YPD medium, before they got transferred onto different growth conditions, and no additional essential genes were detected in those other media, the final list of essential genes applies to all five growth conditions considered.

Combining data about all non-dubious essential and inessential genes, information on the fitness of deletion strains in the five media considered is known for 1404 (536 essential and 868 inessential genes) of the 1487 different genes in the Gavin *et al*. complexes and for 3770 (420 essential and 3350 inessential genes) of the 4408 yeast genes that have not been reported to belong to any macromolecular assembly.

### Partitioning of fitness values into fitness categories

Using a similar approach as Gu *et al*. [47], we partitioned the fitness values into four categories: 'strong negative effect' (*f *< 0.8), 'moderate negative effect' (0.8 ≤ *f *< 0.95), 'weak or no effect' (0.95 ≤ *f *< 1.05) and 'positive effect' (1.05 ≤ *f*), considering essential genes to be part of the 'strong negative effect' category by assigning them a fitness value of zero. We chose the upper threshold for the 'weak or no effect' category symmetrically to the lower threshold, because in this range the data is normally distributed, and we did not want to lose information about gene deletions resulting in a strain fitness better than the pool average.

### Calculation of enrichments

We calculated the enrichments for genes in complexes in the different fitness categories compared to genes not part of complexes by taking the ratio of the fraction of genes which are present in complexes in a given fitness category and the fraction of genes in that same fitness category which are not in complexes, followed by log_2 _-transformation to get a symmetrical range of values. For instance, a ratio of 4 in a given category would thus equal an enrichment of 2, whereas a ratio of 0.25 would equal an enrichment of -2. We used the same calculation of enrichment when comparing the strain fitness upon deletion of genes in cores and genes in attachments and when assessing whether isoform attachments are more likely to show a positive expected fitness effect upon deletion of a random component than isoform cores.

### Computation of p-values

To assess the statistical significance of our findings, we computed p-values using Fisher's exact test. For the computation of a p-value for the correlation coefficient between strain fitness upon deletion of protein-coding genes and protein abundance, we used a Monte Carlo permutation test. This test was based on a random background of 10,000 sets of fitness and abundance annotations, constructed by shuffling the original gene annotations, which ensures that the fitness and abundance distributions remain unchanged. Similarly, we also used a Monte Carlo permutation test to calculate a p-value for the correlation coefficient between the number of isoforms and the number of distinct isoform fitness profiles. This permutation test was based on a random background of 10,000 sets of annotations, shuffling the number of isoforms and the number of distinct isoform fitness profiles for each complex, which retains the distributions of those values. When comparing the average abundance of essential and inessential genes, we employed a Mann-Whitney U test to assess the statistical significance.

### Determination of the coherence of fitness values in a given gene set

We computed the coherence of a given isoform, core, set of attachment proteins, MIPS or Krogan complex based on the raw fitness values, by calculating the fraction of gene pairs in the respective gene set which have a sufficiently small fitness distance. Our definition of "sufficiently small fitness distance" was motivated by the fact that experimental variability has led to slightly different measurements of fitness in the two time-course experiments conducted by Steinmetz *et al*. [3] (see Additional file 1: Fig. S11). To account for this variability, we consider two genes as having highly similar fitness values, if their fitness distance (measured as the Euclidean distance in the fitness space of all five media) is not bigger than the average fitness distance between the time-course one and two measurements plus one standard deviation (to consider the spread of the data). To rationalize this definition, we calculated the following supporting data: First, most pairs of genes with a sufficiently small fitness distance fall into the same fitness category. Depending on the growth condition this means 68% (YPE) to 95% (YPD) of those gene pairs. And second, 82–84% of gene pairs in the same fitness category have a sufficiently small fitness distance according to the above definition, compared to only 43% of all gene pairs. Thus, the fraction of gene pairs in the same fitness category that have a sufficiently small fitness distance is about twice as high as the fraction of all gene pairs. We then defined those sets of genes as coherent, for which more than 2/3 of all gene pairs have a sufficiently small fitness distance.

For comparison, we also computed the coherence of a given isoform, core, set of attachment proteins, MIPS or Krogan complex based on the fitness categories, by calculating for each growth condition the fraction of gene pairs in the respective gene set for which both genes fall into the same fitness category, again defining those gene sets as coherent for which this is the case for more than 2/3 of all gene pairs. We employed both coherence definitions independently and report the ranges of coherent gene sets of each type. In general, the larger any set of genes is, the smaller the probability for the fitness values of its individual gene components to be coherent. As the different types of sets (i.e. isoforms, cores, attachments, MIPS and Krogan complexes) have different distributions of the number of components, to avoid this bias, we first size-corrected the different sets. We created a 'minimal common distribution', which contains the minimum number of gene sets of a given size across all different types of sets. Then, we constructed 1,000 sets of size-corrected isoforms, cores, attachments, MIPS and Krogan complexes based on this 'minimal common distribution', by random sampling from the original sets. Finally, we computed the coherences of those 1,000 sample sets of each type and report the average values.

### Quantification of the fitness of whole isoforms, cores and attachments

To quantify the fitness of a whole isoform, as well as its core and attachments, we used the average fitness value of the given gene set, because it represents the expected fitness of the yeast strain when deleting a random gene of the respective isoform, core or attachments under the assumption that each gene has the same probability for being selected for deletion.

### Computation of correlation coefficients

As neither the number of complex isoforms nor the number of distinct isoform fitness profiles per complex are normally distributed, we did not use the Pearson correlation coefficient. Instead, we employed Goodman and Kruskal's gamma coefficient which is a non-parametric (i.e. distribution-free) measure of correlation based on the difference between the number of concordant and discordant pairs, ignoring ties (which is important here, because many complexes have the same number of isoforms or the same number of distinct isoform fitness profiles). We used the same coefficient for analyzing the correlation between strain fitness upon deletion of protein-coding genes and protein abundance, as determined by Ghaemmaghami *et al*. [30] (see Additional file 3: Protein abundances).

### Identification of duplicate genes in the yeast genome

To identify gene duplicates (i.e. paralogues) in yeast, we performed a BLASTP [48] search of every single yeast gene against the whole yeast genome (5895 non-dubious genes), using an E-value threshold of 10^-10 ^to filter out insignificant results and a coverage threshold of 85% to ensure that a sufficiently large part of the gene could be aligned. We then considered those pairs of genes as duplicates which found each other in the BLASTP search.

## Authors' contributions

RAP, MMB and PA conceived the study and designed the experiments. RAP implemented and performed the experiments. RAP and PA analyzed and interpreted the results. RAP, MMB and PA wrote the manuscript. All authors read and approved the final manuscript.

## Supplementary Material

Additional file 1 Supplementary tables S1 and S2 show the fraction and enrichment in the strong negative effect category of genes in unknown complexes and of unknown genes in complexes, respectively. Supplementary figures S1-6 depict the comparison of the fitness of yeast strains upon deletion of genes in complexes and those not in complexes in all five growth conditions, also when excluding essential genes, as well as for the MIPS and Krogan sets of complexes. Supplementary figure S7 shows the correlation between the number of complexes in which a gene is present and the number of potential interactors, while supplementary figure S8 depicts the fitness of yeast strains in all five growth conditions upon deletion of inessential genes present in multiple complexes. Supplementary figure S9 compares strain fitness upon deletion of protein-coding genes and protein abundance, and supplementary figure S10 compares the fitness of yeast strains upon deletion of genes unique to complex cores and genes unique to attachments in all five growth conditions. Finally, supplementary figure S11 illustrates the similarity of the fitness values for the different yeast deletion strains measured in time-course one and two. Click here for file

Additional file 2 This file provides tables containing the expected fitness effects upon deletion of a random component of the given core or set of attachment proteins for all 5979 isoforms across different growth conditions and the expected fitness effects upon deletion of a random component of whole isoforms, which are visualized in Fig. 5 and Fig. 6, respectively. Click here for file

Additional file 3 This file provides tables containing the set of 491 complexes and the set of 5979 complex isoforms defined by Gavin *et al*. [9], as well as the set of 266 hand-curated yeast complexes in the MIPS database [29] and the set of 547 yeast complexes defined by Krogan *et al*. [10]. This file also provides the quantitative fitness data for 4218 non-dubious inessential genes across two fermentable and three non-fermentable media, measured by Steinmetz *et al*. [3], the confident list of 956 non-dubious essential genes used in this work and the protein abundances determined by Ghaemmaghami *et al*. [30]. Click here for file
