# Supplementary material for: Exploiting gene deletion fitness effects in yeast to understand the modular architecture of protein complexes under different growth conditions
Source: BMC Syst Biol. 2009 Jul 18;3:74. doi: 10.1186/1752-0509-3-74 (PMC2717920; doi:10.1186/1752-0509-3-74)
Supplement: Additional file 1 — Supplementary tables S1 and S2 show the fraction and enrichment in the strong negative effect category of genes in unknown complexes and of unknown genes in complexes, respectively. Supplementary figures S1-6 depict the comparison of the fitness of yeast strains upon deletion of genes in complexes and those not in complexes in all five growth conditions, also when excluding essential genes, as well as for the MIPS and Krogan sets of complexes. Supplementary figure S7 shows the correlation between the number of complexes in which a gene is present and the number of potential interactors, while supplementary figure S8 depicts the fitness of yeast strains in all five growth conditions upon deletion of inessential genes present in multiple complexes. Supplementary figure S9 compares strain fitness upon deletion of protein-coding genes and protein abundance, and supplementary figure S10 compares the fitness of yeast strains upon deletion of genes unique to complex cores and genes unique to attachments in all five growth conditions. Finally, supplementary figure S11 illustrates the similarity of the fitness values for the different yeast deletion strains measured in time-course one and two. [file 1752-0509-3-74-S1.pdf]

Supplementary Information

Exploiting gene deletion fitness effects in yeast to understand the modular architecture of protein complexes under different growth conditions

Roland A. Pache[1,2], M. Madan Babu[3,*] and Patrick Aloy[1,2,4,*]

1. Structural and Computational Biology, Institute for Research in Biomedicine (IRB) Barcelona, c/ Baldiri Reixac 10-12, 08028 Barcelona, Spain
2. Life Sciences, Barcelona Supercomputing Center (BSC), c/ Jordi Girona 29, 08034 Barcelona, Spain
3. Systems Biology, MRC Laboratory of Molecular Biology, Hills Road, CB2 2QH Cambridge, UK
4. Institució Catalana de Recerca i Estudis Avançats (ICREA), Passeig Lluís Companys 23, 08010 Barcelona, Spain

* Corresponding authors:
M. Madan Babu. Tel: +44 1223402208; Email: madanm@mrc-lmb.cam.ac.uk
Patrick Aloy. Tel: +34 934039690; Fax: +34 934039954; Email: patrick.aloy@irbbarcelona.org

1 Supplementary Tables

	Fermentable				Non-fermentable					
	YPD		YPDGE		YPG		YPE		YPL	
	(i)	(ii)	(i)	(ii)	(i)	(ii)	(i)	(ii)	(i)	(ii)
Genes in complexes	49.5%	19.4%	49.9%	20.1%	48.7%	18.1%	50.2%	20.4%	47.1%	15.6%
Other genes	19.9%	6.7%	20.4%	7.2%	22.3%	9.5%	22.8%	10.0%	21.5%	8.5%
Enrichment	1.32	1.54	1.30	1.47	1.13	0.93	1.14	1.02	1.13	0.87
P-value	$1.66 \cdot 10^{-73}$	$1.11 \cdot 10^{-20}$	$1.30 \cdot 10^{-72}$	$4.14 \cdot 10^{-20}$	$2.63 \cdot 10^{-57}$	$2.09 \cdot 10^{-9}$	$1.28 \cdot 10^{-60}$	$4.77 \cdot 10^{-12}$	$3.56 \cdot 10^{-55}$	$1.88 \cdot 10^{-7}$

Table S1: ***Fraction and enrichment of genes in unknown complexes in the strong negative effect category*** Fraction and enrichment of genes in complexes compared to genes not in complexes in the strong negative fitness effect category, excluding all those complexes with a significant overlap to known complexes in the hand-curated MIPS database [1]. (i) essential and inessential genes, (ii) only inessential genes. P-values were computed using a one-sided Fisher's exact test. Enrichments are given on a $\log_2$-scale.

	Fermentable				Non-fermentable					
	YPD		YPDGE		YPG		YPE		YPL	
	(i)	(ii)	(i)	(ii)	(i)	(ii)	(i)	(ii)	(i)	(ii)
Genes in complexes	21.9%	8.1%	21.9%	8.1%	20.5%	6.5%	21.9%	8.1%	21.9%	8.1%
Other genes	6.0%	2.1%	6.2%	2.4%	6.8%	3.0%	6.9%	3.1%	7.1%	3.3%
Enrichment	1.88	1.91	1.83	1.78	1.60	1.11	1.67	1.38	1.63	1.28
P-value	$1.71 \cdot 10^{-5}$	$1.60 \cdot 10^{-2}$	$2.46 \cdot 10^{-5}$	$2.20 \cdot 10^{-2}$	$2.26 \cdot 10^{-4}$	$1.32 \cdot 10^{-1}$	$7.94 \cdot 10^{-5}$	$5.42 \cdot 10^{-2}$	$1.08 \cdot 10^{-4}$	$6.68 \cdot 10^{-2}$

Table S2: ***Fraction and enrichment of unknown genes in complexes in the strong negative effect category*** Fraction and enrichment of genes in complexes compared to genes not in complexes in the strong negative fitness effect category, considering only those genes annotated as 'Uncharacterized ORF'. (i) essential and inessential genes, (ii) only inessential genes. P-values were computed using a one-sided Fisher's exact test. Enrichments are given on a $\log_2$-scale.

2 Supplementary Figures

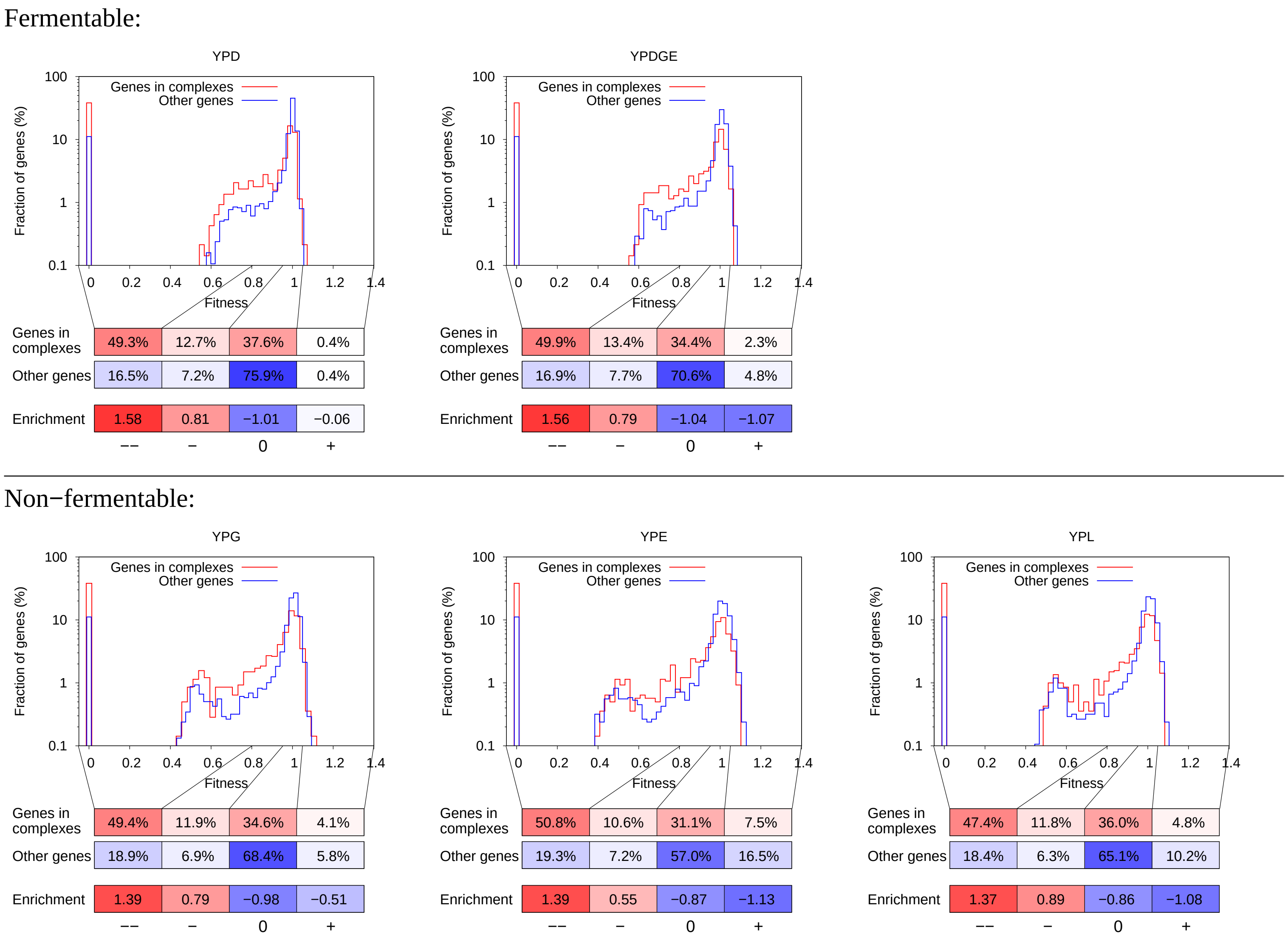

Figure S1: ***Comparison of the fitness of yeast strains upon deletion of genes in complexes and those not in complexes***
Distributions of strain fitness upon deletion of genes in complexes (red) and genes not part of complexes (blue) in the two fermentable and the three non-fermentable media considered. Genes with a fitness of zero are essential. The fitness values of individual genes are partitioned into four categories: 'strong negative effect' (--), 'moderate negative effect' (-), 'weak or no effect' (0) and 'positive effect' (+). Different shades of red illustrate the percentage of genes in complexes (for which we have essentiality data) in the four fitness categories, with deep red corresponding to 100% (1404 genes). Different shades of blue illustrate the percentage of genes not in complexes (for which we have essentiality data) in the four fitness categories, with deep blue corresponding to 100% (3770 genes). Enrichments are given on a $\log_2$-scale.

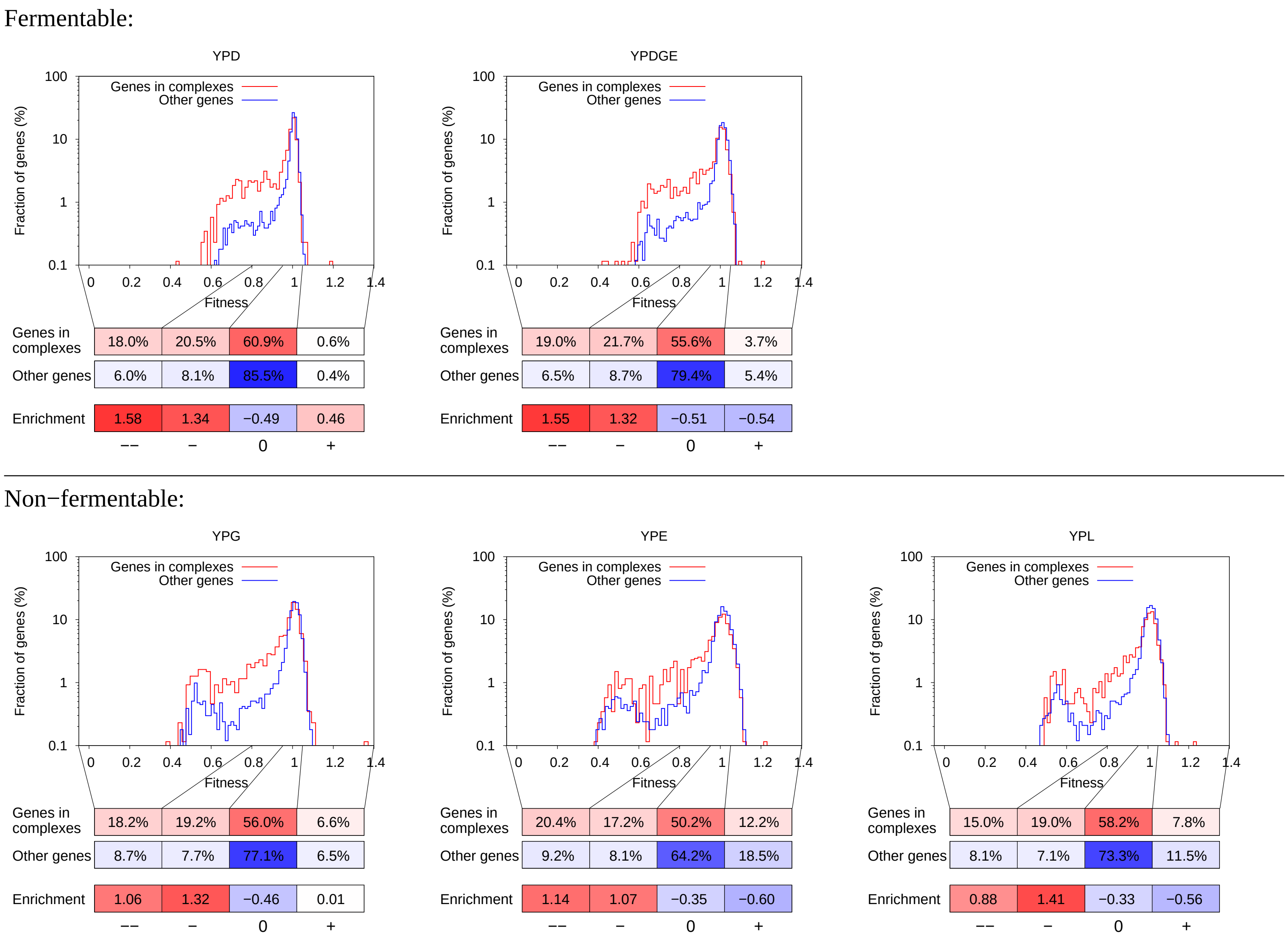

Figure S2: ***Comparison of the fitness of yeast strains upon deletion of inessential genes in complexes and those not in complexes***
Distributions of strain fitness upon deletion of inessential genes in complexes (red) and inessential genes not part of complexes (blue) in the two fermentable and the three non-fermentable media considered. The fitness values of individual genes are partitioned into four categories: ’strong negative effect’ (--), ’moderate negative effect’ (-), ’weak or no effect’ (0) and ’positive effect’ (+). Different shades of red illustrate the percentage of genes in complexes (for which we have essentiality data) in the four fitness categories, with deep red corresponding to 100% (868 genes). Different shades of blue illustrate the percentage of genes not in complexes (for which we have essentiality data) in the four fitness categories, with deep blue corresponding to 100% (3350 genes). Enrichments are given on a $\log_2$-scale.

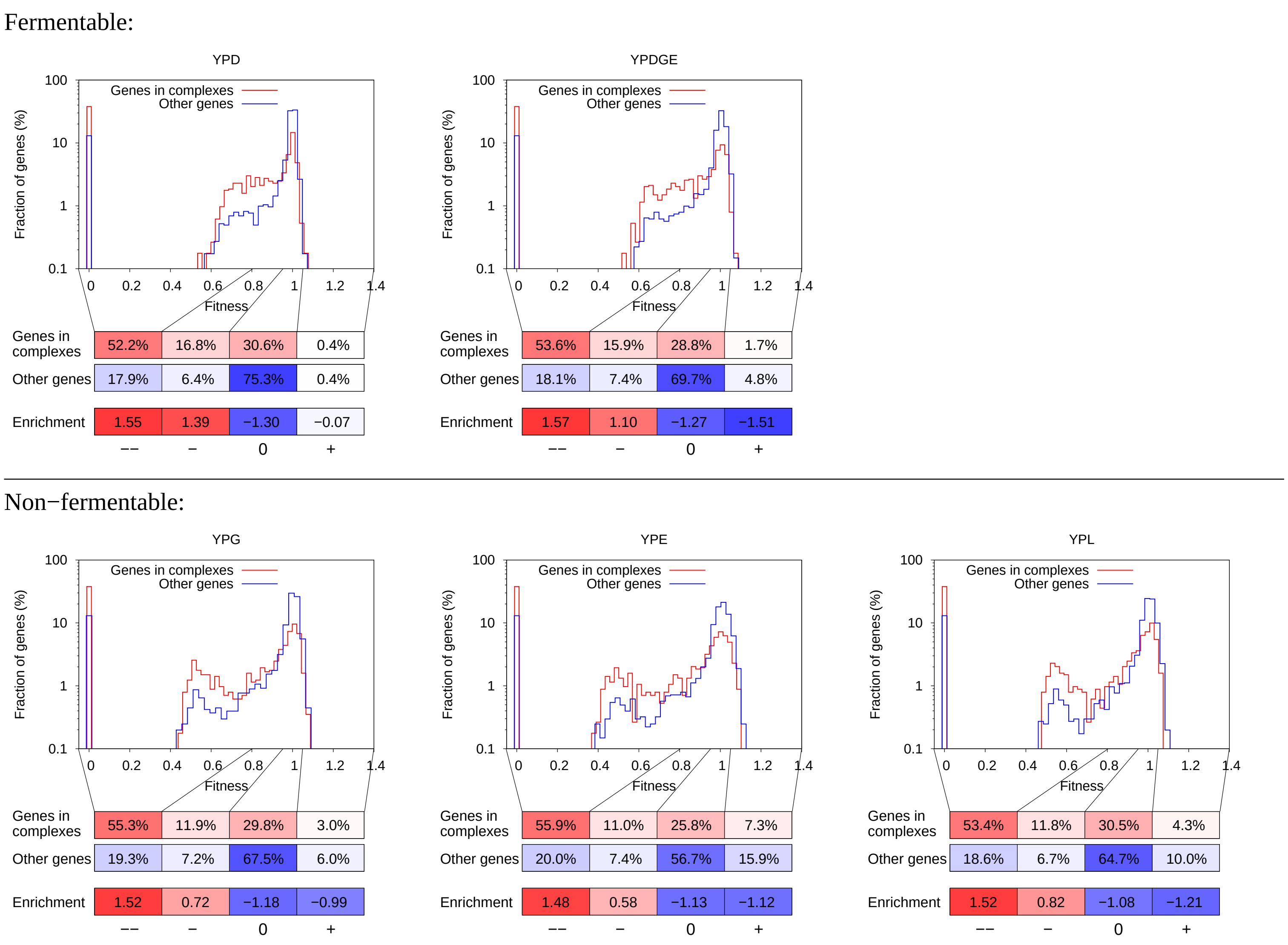

Figure S3: ***Comparison of the fitness of yeast strains upon deletion of genes in MIPS complexes and those not in MIPS complexes***
Distributions of strain fitness upon deletion of genes in the hand-curated set of 266 yeast complexes in the Munich Information Center for Protein Sequences (MIPS) database [1] (red) and genes not part of MIPS complexes (blue) in the two fermentable and the three non-fermentable media considered. Genes with a fitness of zero are essential. The fitness values of individual genes are partitioned into four categories: ’strong negative effect’ (--), ’moderate negative effect’ (-), ’weak or no effect’ (0) and ’positive effect’ (+). Different shades of red illustrate the percentage of genes in complexes (for which we have essentiality data) in the four fitness categories, with deep red corresponding to 100% (1134 genes). Different shades of blue illustrate the percentage of genes not in complexes (for which we have essentiality data) in the four fitness categories, with deep blue corresponding to 100% (4040 genes). Enrichments are given on a $\log_2$-scale.

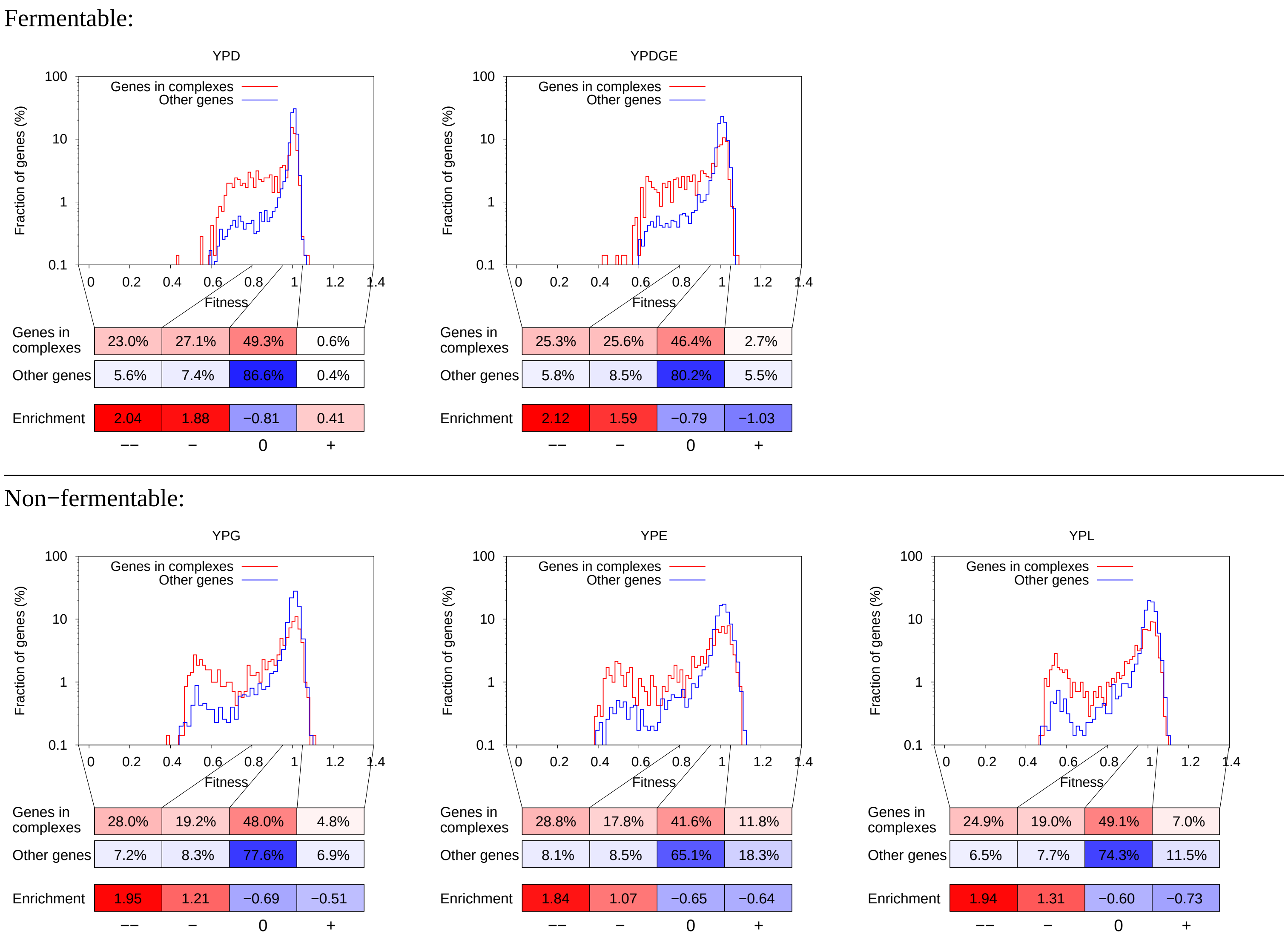

Figure S4: ***Comparison of the fitness of yeast strains upon deletion of inessential genes in MIPS complexes and those not in MIPS complexes*** Distributions of strain fitness upon deletion of inessential genes in the hand-curated set of 266 yeast complexes in the Munich Information Center for Protein Sequences (MIPS) database [1] (red) and inessential genes not part of MIPS complexes (blue) in the two fermentable and the three non-fermentable media considered. The fitness values of individual genes are partitioned into four categories: 'strong negative effect' (--), 'moderate negative effect' (-), 'weak or no effect' (0) and 'positive effect' (+). Different shades of red illustrate the percentage of genes in complexes (for which we have essentiality data) in the four fitness categories, with deep red corresponding to 100% (704 genes). Different shades of blue illustrate the percentage of genes not in complexes (for which we have essentiality data) in the four fitness categories, with deep blue corresponding to 100% (3514 genes). Enrichments are given on a $\log_2$-scale.

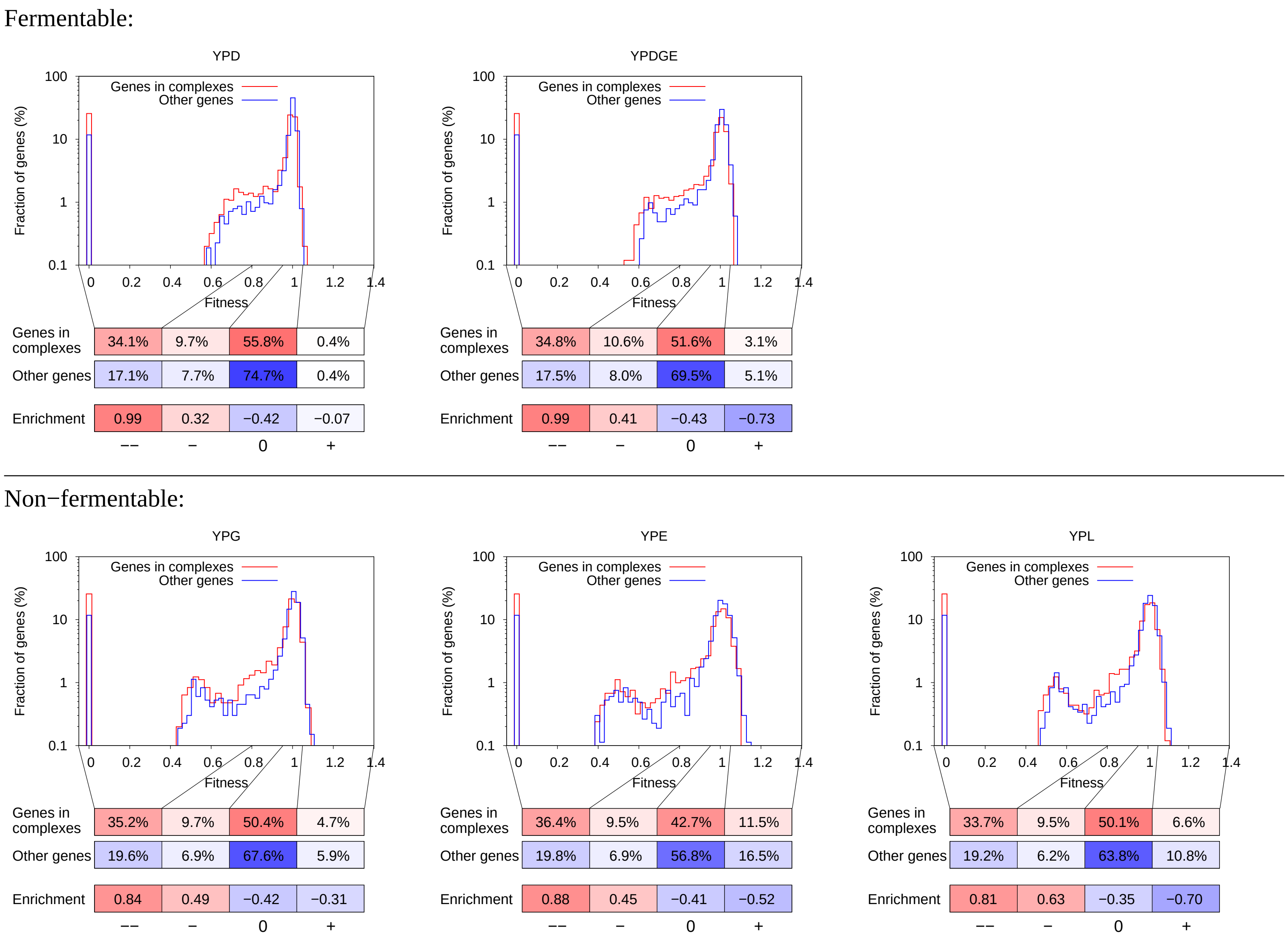

Figure S5: ***Comparison of the fitness of yeast strains upon deletion of genes in Krogan complexes and those not in Krogan complexes***
Distributions of strain fitness upon deletion of genes in the set of 547 yeast complexes defined by Krogan *et al.* [2] (red) and genes not part of Krogan complexes (blue) in the two fermentable and the three non-fermentable media considered. Genes with a fitness of zero are essential. The fitness values of individual genes are partitioned into four categories: 'strong negative effect' (--), 'moderate negative effect' (-), 'weak or no effect' (0) and 'positive effect' (+). Different shades of red illustrate the percentage of genes in complexes (for which we have essentiality data) in the four fitness categories, with deep red corresponding to 100% (2515 genes). Different shades of blue illustrate the percentage of genes not in complexes (for which we have essentiality data) in the four fitness categories, with deep blue corresponding to 100% (2659 genes). Enrichments are given on a $\log_2$-scale.

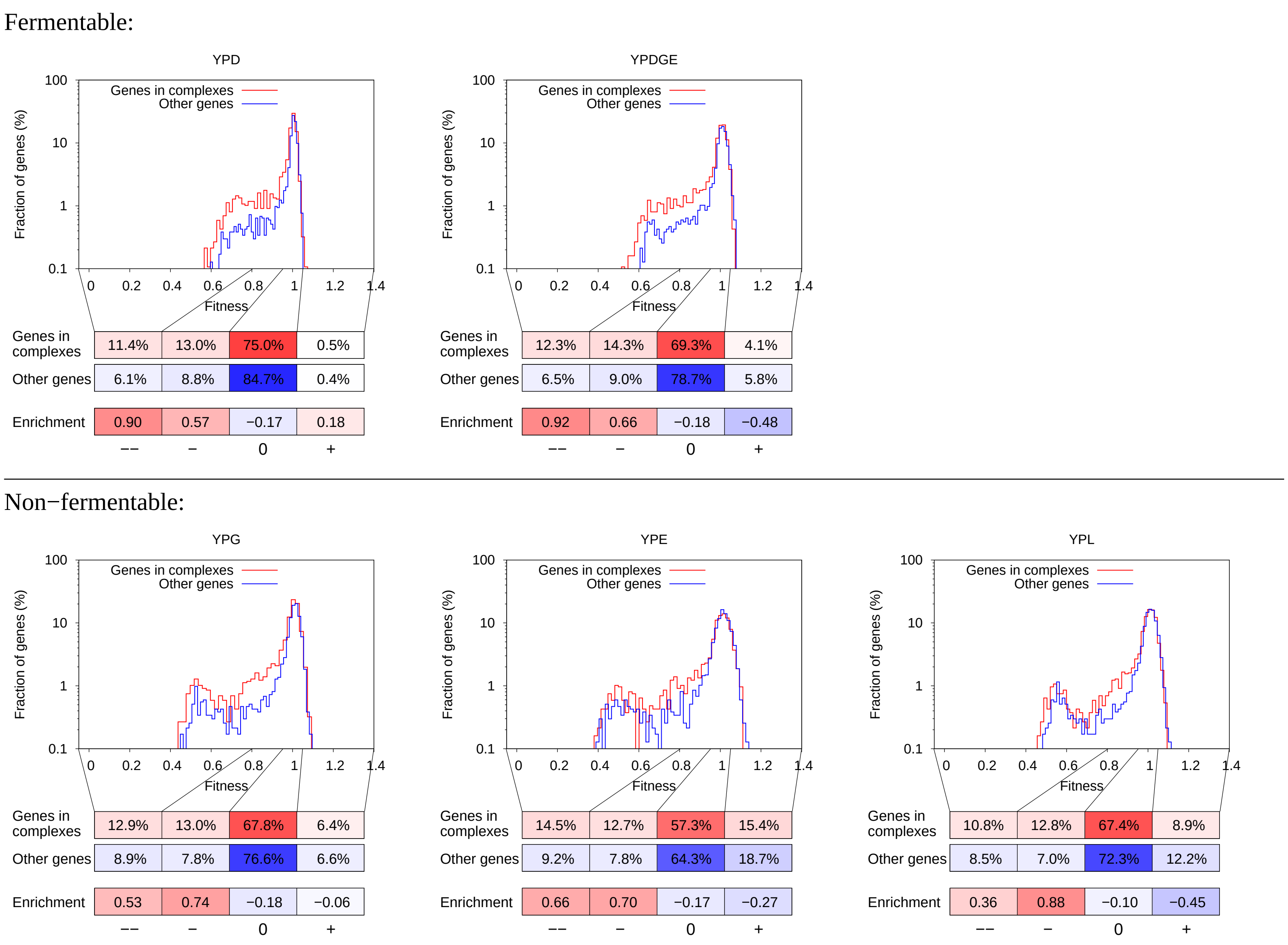

Figure S6: ***Comparison of the fitness of yeast strains upon deletion of inessential genes in Krogan complexes and those not in Krogan complexes*** Distributions of strain fitness upon deletion of inessential genes in the set of 547 yeast complexes defined by Krogan *et al.* [2] (red) and inessential genes not part of Krogan complexes (blue) in the two fermentable and the three non-fermentable media considered. The fitness values of individual genes are partitioned into four categories: ’strong negative effect’ (--), ’moderate negative effect’ (-), ’weak or no effect’ (0) and ’positive effect’ (+). Different shades of red illustrate the percentage of genes in complexes (for which we have essentiality data) in the four fitness categories, with deep red corresponding to 100% (1871 genes). Different shades of blue illustrate the percentage of genes not in complexes (for which we have essentiality data) in the four fitness categories, with deep blue corresponding to 100% (2347 genes). Enrichments are given on a $\log_2$-scale.

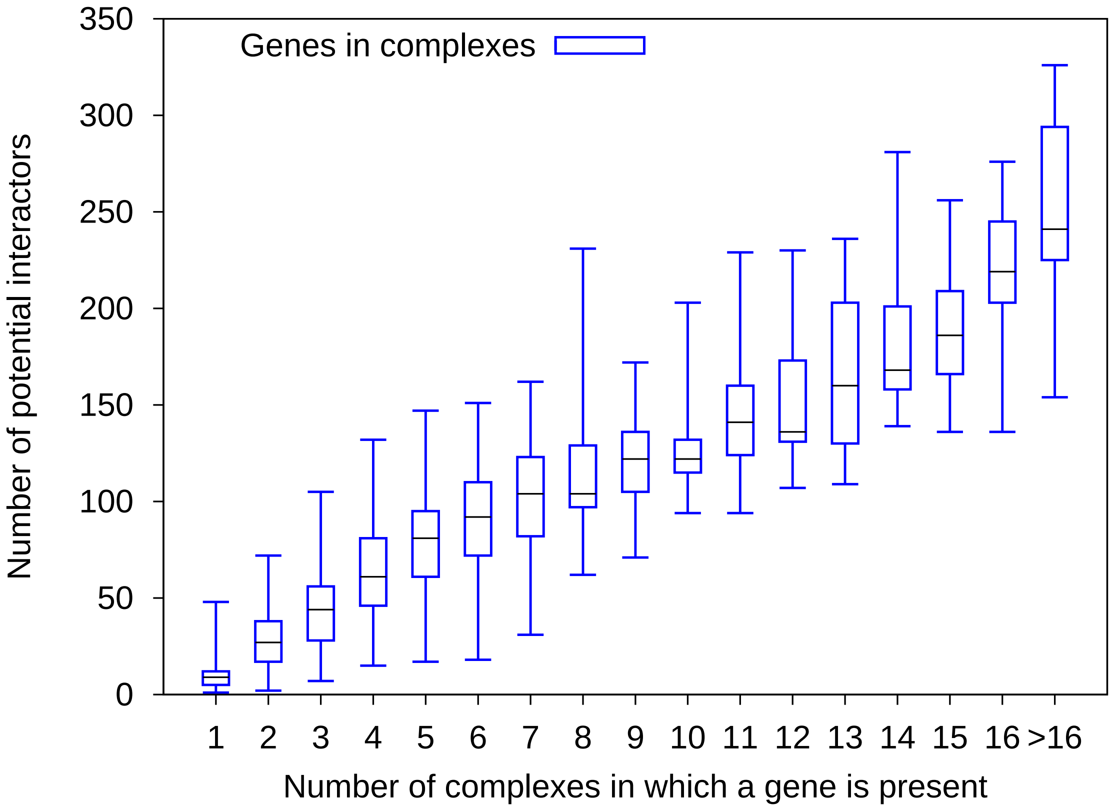

Figure S7: ***Comparison of the number of complexes in which a gene is present and the number of potential interactors***
Box-and-whisker plots of the number of potential interactors of genes present in multiple complexes, based on the matrix model [3] which assumes that each protein interacts with each other protein in a complex. Start and end of the boxes indicate the first and third quartile of the number of interactors distribution of genes present in a given number of complexes, and whiskers denote the respective minimum and maximum number of interactors. The medians of the respective distributions are shown as black bars. As only 21 genes are present in more than 16 complexes, we grouped them together. The gamma correlation coefficient between the number of complexes in which a gene is present and its number of potential interactors is 0.83.

Fermentable:

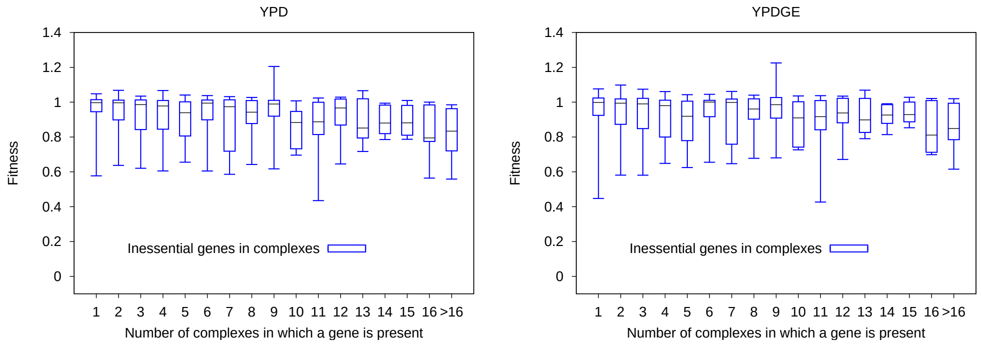

Non−fermentable:

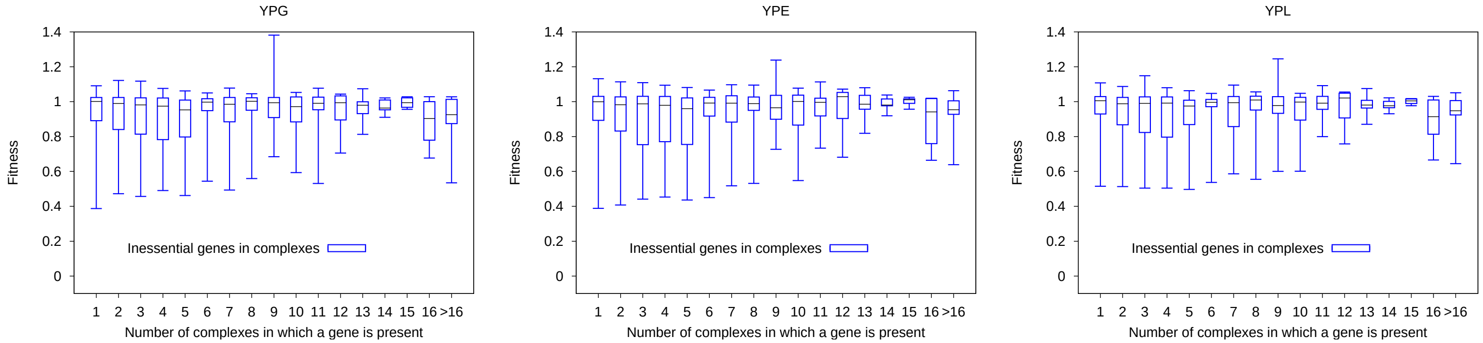

Figure S8: ***Fitness of yeast strains upon deletion of inessential genes present in multiple complexes***
Box-and-whisker plots of strain fitness upon deletion of inessential genes which are part of multiple complexes, measured in the two fermentable and the three non-fermentable media considered. Start and end of the boxes indicate the first and third quartile of the fitness distribution of inessential genes present in a given number of complexes, and whiskers denote the respective minimum and maximum fitness values. The medians of the respective distributions are shown as black bars. As only 21 inessential genes are present in more than 16 complexes, we grouped them together.

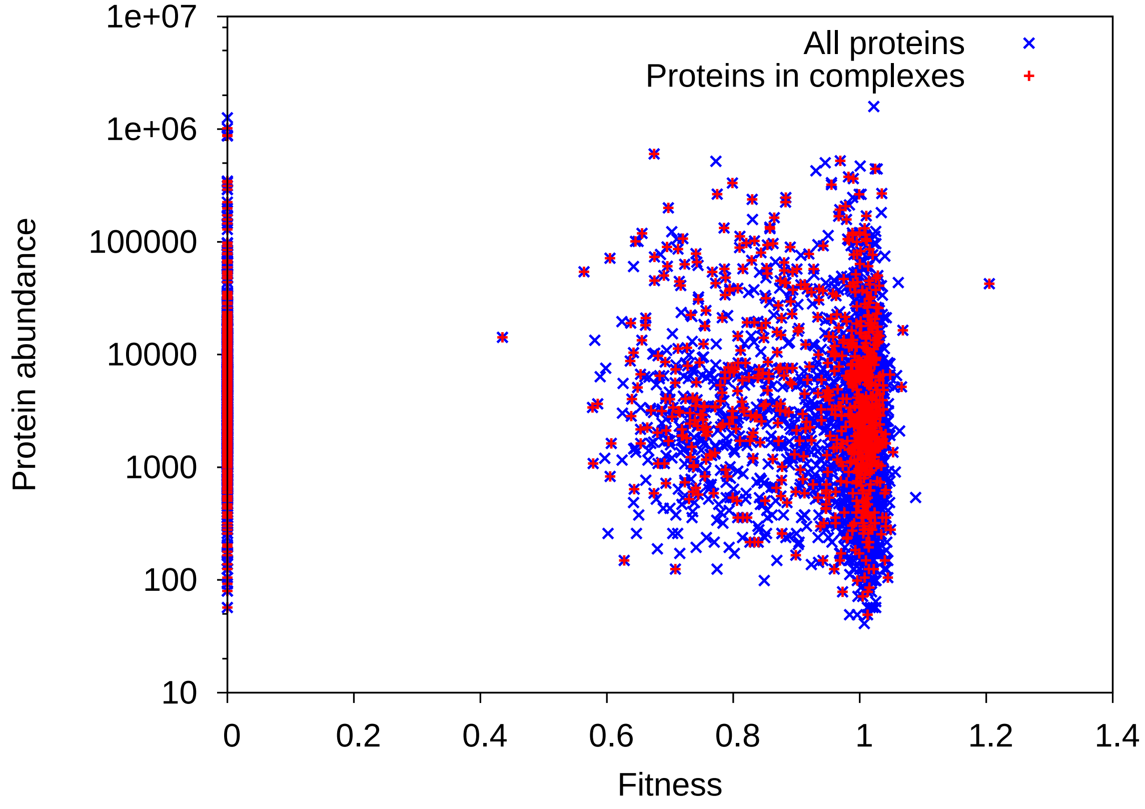

Figure S9: ***Correlation analysis of strain fitness and protein abundance***
Correlation analysis of strain fitness upon deletion of protein-coding genes and protein abundance, comparing all yeast proteins (blue) to only those present in complexes (red). Essential genes are assigned a fitness of 0. Abundances are given on a logarithmic scale.

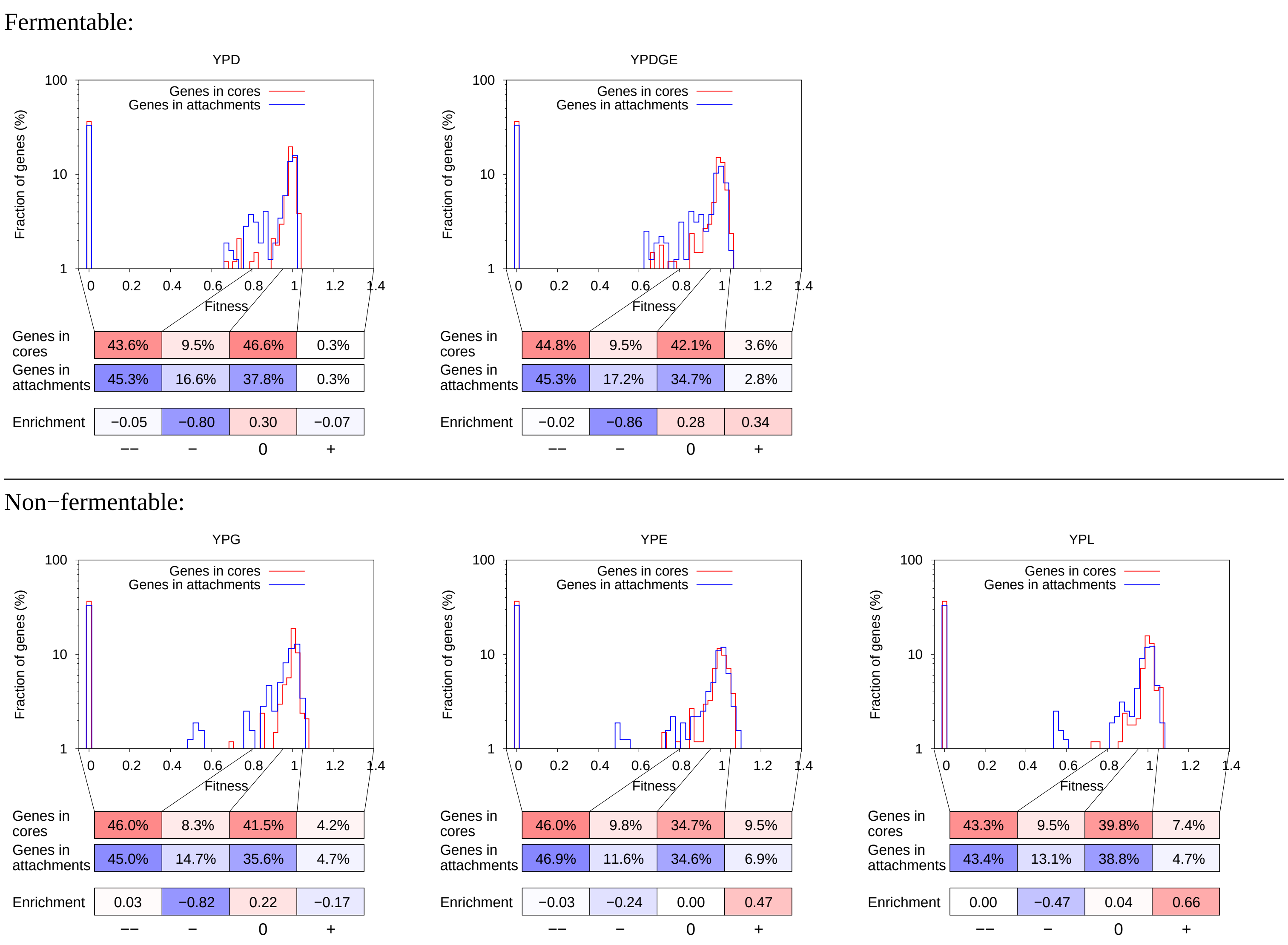

Figure S10: ***Comparison of the fitness of yeast strains upon deletion of genes unique to complex cores and genes unique to attachments***
Distributions of strain fitness upon deletion of genes only present in cores (red) and genes only present in attachments (blue) in the two fermentable and the three non-fermentable media considered. Genes with a fitness of zero are essential. The fitness values of individual genes are partitioned into four categories: ’strong negative effect’ (--), ’moderate negative effect’ (-), ’weak or no effect’ (0) and ’positive effect’ (+). Different shades of red illustrate the percentage of genes only present in cores (for which we have essentiality data) in the four fitness categories, with deep red corresponding to 100% (337 genes). Different shades of blue illustrate the percentage of genes only present in attachments (for which we have essentiality data) in the four fitness categories, with deep blue corresponding to 100% (320 genes). Enrichments are given on a $\log_2$-scale.

Fermentable:

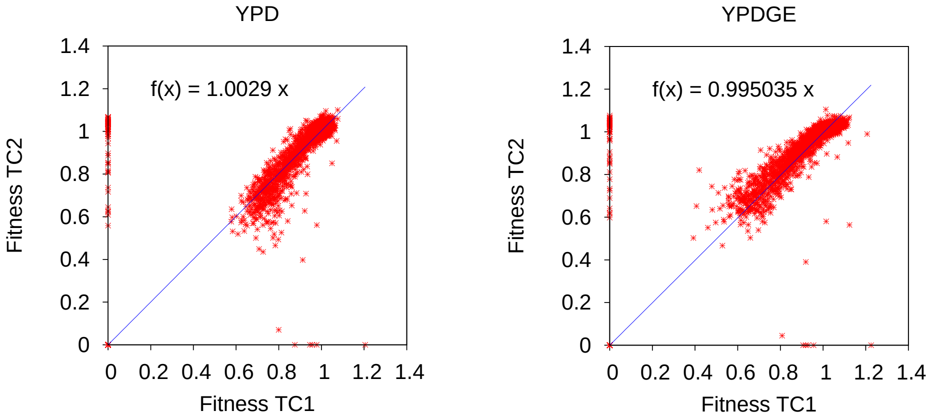

Non−fermentable:

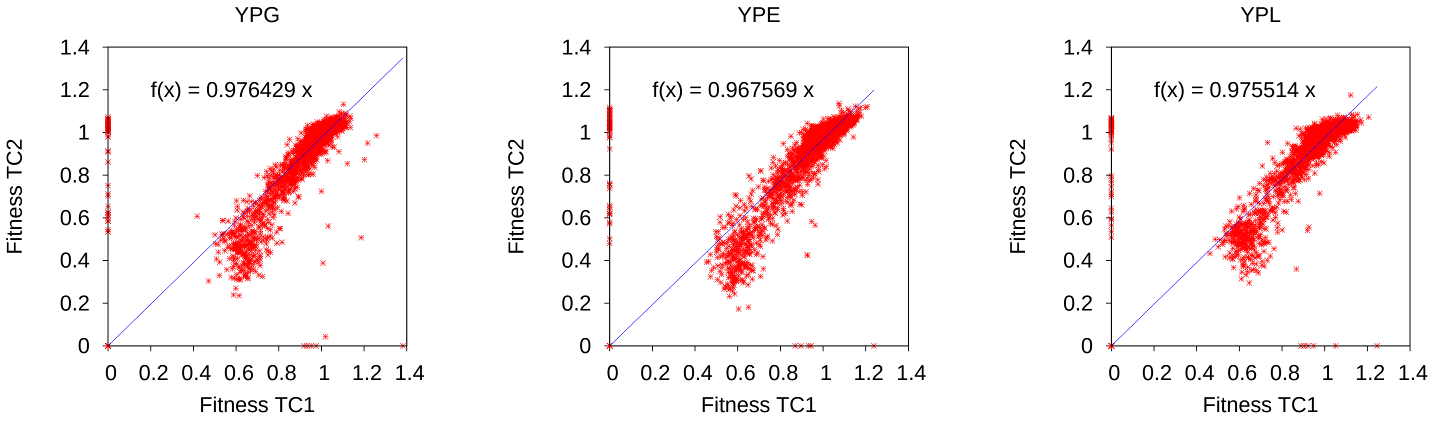

Figure S11: ***Comparison of the fitness values for the different yeast deletion strains measured in time-course one and two***
Comparison of the fitness of yeast deletion strains measured in the two time-course experiments conducted by Steinmetz *et al.* [4] in the two fermentable and the three non-fermentable media considered. Fitness TC1: fitness values reported in time-course one, Fitness TC2: fitness values reported in time-course two. The blue line indicates the linear fit and the equations show the respective slope of the fitted line.

Supplementary references

1. Güldener U, Münsterkötter M, Oesterheld M, Pagel P, Ruepp A, Mewes HW, Stümpflen V: **MPact: the MIPS protein interaction resource on yeast.** *Nucleic Acids Res* 2006, **34**(Database issue):D436–D441.

2. Krogan NJ, Cagney G, Yu H, Zhong G, Guo X, Ignatchenko A, Li J, Pu S, Datta N, Tikuisis AP, Punna T, PeregrÃn-Alvarez JM, Shales M, Zhang X, Davey M, Robinson MD, Paccanaro A, Bray JE, Sheung A, Beattie B, Richards DP, Canadien V, Lalev A, Mena F, Wong P, Starostine A, Canete MM, Vlasblom J, Wu S, Orsi C, Collins SR, Chandran S, Haw R, Rilstone JJ, Gandi K, Thompson NJ, Musso G, Onge PS, Ghanny S, Lam MHY, Butland G, Altaf-Ul AM, Kanaya S, Shilatifard A, O'Shea E, Weissman JS, Ingles CJ, Hughes TR, Parkinson J, Gerstein M, Wodak SJ, Emili A, Greenblatt JF: **Global landscape of protein complexes in the yeast Saccharomyces cerevisiae.** *Nature* 2006, **440**(7084):637–643.

3. Bader GD, Hogue CWV: **Analyzing yeast protein-protein interaction data obtained from different sources.** *Nat Biotechnol* 2002, **20**(10):991–997.

4. Steinmetz LM, Scharfe C, Deutschbauer AM, Mokranjac D, Herman ZS, Jones T, Chu AM, Giaever G, Prokisch H, Oefner PJ, Davis RW: **Systematic screen for human disease genes in yeast.** *Nat Genet* 2002, **31**(4):400–404.
